# Navitoclax-encapsulated dual pH-responsive copolymeric nanoparticles improves hepatocellular carcinoma treatment with induction of apoptosis and immune activation

**DOI:** 10.1186/s12951-026-04733-3

**Published:** 2026-07-14

**Authors:** Jiahao Wang, Tianao Xie, Heng Jiang, Yukai Shan, Sarun Juengpanich, Shijie Li, Win Topatana, Yeling Liu, Hong Wan, Yeben Qian, Peng Chen, Bo Chen

**Affiliations:** 1 Department of General Surgery, The First Affiliated Hospital of Anhui Medical University, Hefei, 230022 China; 2 Department of General Surgery, Sir Run Run Shaw Hospital, School of Medicine, Zhejiang University, Hangzhou, 310000 China; 3 Department of Rheumatology and Clinical Immunology, Peking Union Medical College Hospital, Beijing, 100191 China; 4 Department of General Surgery, The Second Affiliated Hospital of Anhui Medical University, Hefei, 230601 China

**Keywords:** Nnavitoclax, Hepatocellular carcinoma, Reactive oxygen species, Immunogenic cell death

## Abstract

**Background:**

Navitoclax (ABT-263) exhibits strong cytotoxic effects against hepatocellular carcinoma cells in vitro; however, its therapeutic efficacy in vivo is limited by insufficient local drug accumulation and compensatory upregulation of the anti-apoptotic protein myeloid cell leukemia-1 during long-term treatment. In addition, effective immune activation in hepatocellular carcinoma remains challenging due to the immune-privileged hepatic microenvironment. This study aimed to overcome these limitations through a tumor-targeted nanotherapeutic strategy.

**Results:**

Dual pH-responsive copolymeric nanoparticles modified with folic acid and co-loaded with navitoclax and indocyanine green (FPN_AI_) were developed. FPN_AI_ preferentially accumulated at hepatocellular carcinoma sites through folate receptor–mediated targeting and released navitoclax in response to the acidic tumor microenvironment, thereby inducing mitochondria-mediated apoptosis. Upon near-infrared irradiation, indocyanine green generated excessive reactive oxygen species, leading to downregulation of myeloid cell leukemia-1 and further enhancement of apoptotic signaling. The combined effects of navitoclax and reactive oxygen species synergistically promoted tumor cell apoptosis. Moreover, apoptosis induced by FPN_AI_ triggered immunogenic cell death, characterized by dendritic cell maturation and activation of cytotoxic CD8⁺ T cells. In vivo studies demonstrated that FPN_AI_ with near-infrared irradiation achieved significantly improved antitumor efficacy compared with navitoclax monotherapy, while maintaining favorable tumor-targeting capability and biosafety.

**Conclusions:**

This study demonstrates that FPN_AI_ enhances the antitumor activity of navitoclax by integrating targeted drug delivery, reactive oxygen species–mediated mitochondrial apoptosis, and immunogenic cell death induction. The proposed nanotherapeutic platform represents a promising approach for improving hepatocellular carcinoma treatment outcomes.

**Graphical Abstract:**

**Figure:**
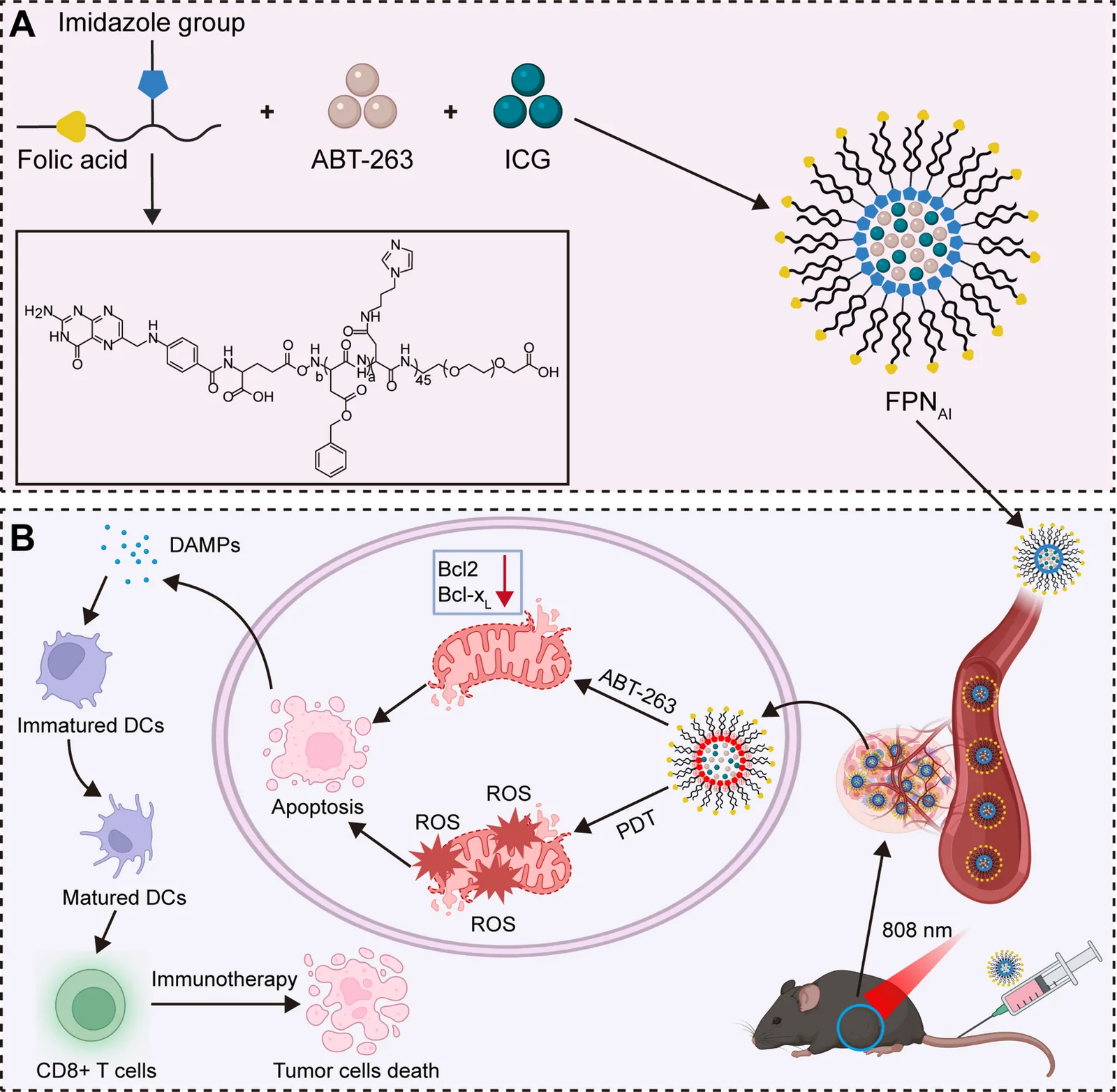

**Supplementary Information:**

The online version contains supplementary material available at https://doi.org/10.1186/s12951-026-04733-3.

## Introduction

Hepatocellular carcinoma (HCC) ranks among the top causes of cancer-related deaths with an increasing annual incidence rate in developing countries [1–3]. Surgery is the main curative treatment option for HCC, and the 5-year overall survival rate after operation reaches about 70% [4]. However, only early-stage patients are eligible for surgery and nearly 80% patients face recurrence after operation [4]. Other treatment options like trans-arterial chemoembolization (TACE) offer limited benefits to inoperable HCC patients, highlighting the necessity for novel alternative strategies [4, 5].

Recent molecular targeting drugs show promise as supplementary methods for HCC. Navitoclax (ABT-263), a small-molecule Bcl-2 homology 3 mimetic, can specifically binds anti-apoptotic Bcl2 family proteins Bcl-2 and Bcl-xL, liberating the sequestered pro-apoptotic proteins Bax and Bak, thereby activating caspase cascade and ultimately inducing mitochondria-mediated apoptosis [6, 7]. Unlike conventional cytotoxic agents that trigger apoptosis through upstream cellular damage, ABT-263 directly targets anti-apoptotic Bcl-2 family proteins and therefore provides a mechanism-based strategy to restore intrinsic apoptosis in apoptosis-resistant tumor cells. The efficacy and safety of ABT-263 have been validated in hematological tumors via clinical trails, suggesting potential pan-cancer utility [8, 9]. Actually, its cytotoxic effect on solid tumors has also been confirmed in vitro [10–12]. However, the pro-apoptotic effect of ABT-263 in HCC is limited in vivo [13–15]. Two reasons lead to this unsatisfactory outcome. First, long-term application of ABT-263 up-regulates anti-apoptotic protein Mcl-1. Second, relatively insufficient drug concentration occurs at the HCC site when limited dose of ABT-263 is administered. These characteristics provided the rationale for combining ABT-263 with an ICG-mediated PDT module, in which NIR-triggered ROS could provide an additional pro-apoptotic stimulus and potentially attenuate compensatory anti-apoptotic signaling. Regarding the first reason, several methods have been proposed, such as using aspirin or inhibitors of ERK, JNK, AKT or Mcl-1 in combination to counteract Mcl-1 over-expression, and the outcomes are encouraging [13–15]. Regarding the second reason, loading ABT-263 with nanoparticles (NPs) is a feasible approach, which can improve the concentration of ABT-263 at the tumor site [16–18]. Therefore, addressing the two factors is a feasible approach to enhancing both its effectiveness and safety. However, the effectiveness and safety of nanoparticles loading ABT-263 have not been assessed in HCC, and limited researches have been conducted to explore the value of ABT-263 combined with other therapies in treating solid tumors.

HCC faces therapeutic challenges in responding to immune checkpoint blockade therapy with only 15% patients exhibit an objective response, primarily due to the unique immune privilege properties of liver [19, 20]. Recent studies have illustrated that immunogenic cell death (ICD) can remodel this special micro-environment of liver. ICD refers to the release of damage associated molecular patterns (DAMPs) from apoptotic or dead tumor cells. These DAMPs promote dendritic cells (DCs) maturation, thereby inducing the differentiation of T cells into cytotoxic T lymphocytes (CTLs) that combat tumor cells [21–23]. However, conventional ICD inducers lack tumor-targeting specificity, leading to systemic toxicity and low hepatic enrichment efficiency.

To overcome the limitations of ABT-263 and ICD inducers for HCC, we constructed folic acid (FA)-modified copolymeric NPs (FPN_AI_) co-loaded with ABT-263 and indocyanine green (ICG), featuring pH-responsiveness and near infrared light (NIR)-activability. Theoretically, FPN_AI_ can target release ABT-263 and ICG in HCC tissues, trigger ROS generation, and induce mitochondrial apoptosis in HCC cells, ultimately result in immune activation. This combined effect dramatically enhanced the efficacy of HCC therapy. In conclusion, the development of FPN_AI_ illustrated a promising method for HCC treatment via combining apoptosis and immune activation.

## Materials and methods

### Synthesis of PEG-p(API-ASP)-FA

The synthesis of copolymer was based on β-Benzyl-ʟ-aspartate N-carboxy anhydride (BLA-NCA) as a template. BLA-NCA was synthesised by the Fuchs-Farthing method with triphosgene and tetrahydrofuran (THF) (Aladdin Co., Ltd, Shanghai, China) [24, 25]. To prepare poly(ethylene glycol) (PEG)-PBLA, BLA-NCA (3.0 g) was dissolved in a mixture of dichloromethane (CH_2_Cl_2_) (50 mL) (Aladdin Co., Ltd, Shanghai, China) and N, N-dimethylformamide (DMF) (20 mL) (Sigma-Aldrich Co., Ltd, MO, USA) was polymerized using amino PEG acid (0.24 g) (Aladdin Co., Ltd, Shanghai, China) as an initiator. The reaction mixture was stirred at 40℃ in an oil bath for 48 h under argon gas. After 48 h, the PEG-PBLA was purified with diethyl ether and dired under vacuum. To synthesized PEG-PBLA-FA, FA (13.2 mg) (Macklin Co., Ltd, China), N-hydroxysuccinimide (NHS) (3.3 mg) (Aladdin Co., Ltd, Shanghai, China) and 1-(3-Dimethylaminopropyl)-3-ethylcarbodiimide hydrochloride (EDC) (4.6 mg) (Aladdin Co., Ltd, Shanghai, China) were dissolved into DMSO (10 mL) and stirred at room temperature over night under argon gas. The next day, PEG-PBLA (0.4 g) was added to the solution and the reaction mixture was stirred in the same conditions for 24 h. PEG-PBLA-FA was purified and obtained by dialysis (MWCO: 7000Da) and lyophilization. PEG-p(API-ASP)-FA was synthesized by acylation of carboxyl and amino. Briefly, PEG-PBLA-FA (0.2 g) was dissolved in DMSO (5 mL), followed by reacting with 1-(3-aminopropyl)imidazole (API) (0.2 g) at room temperature for 24 h under argon gas. After 24 h, the reaction mixture was drop wisely added into a cooled 0.1 M HCL (20 mL) and stirred 5 min. The final solution was dialyzed against 0.01 M HCL for 24 h (MWCO: 1000 Da) and lyophilized to obtain PEG-p(API-ASP)-FA.

### Synthesis of FPN_AI_

Briefly, PEG-p(API-ASP)-FA (10 mg), ABT-263 (5 mg) (MCE Co., Ltd, NJ, USA) and ICG (5 mg) (Macklin Co., Ltd, China) were dissolved in methanol (5 mL) and dried into a film via rotary evaporation at 60℃ under vacuum. Then, PBS (10 mL, pH = 7.4) was added into the round-bottom flask. After 10 min ultrasonication by a probe sonicator, the reaction mixture was stirred at 60℃ in an oil bath for 6 h. Finally, the solution was dialyzed against PBS (pH = 7.4) (MWCO: 3500 Da) for 24 h and centrifuged three times via ultrafiltration centrifuge tube (10 KDa) (10000 g, 10 min) to remove unloaded ABT-263 and ICG. The FPN_AI_ was dispersed in PBS (10 mL, pH = 7.4) for subsequent studies.

### Characterization of FPN_AI_

Transmission electron microscope (TEM) was applied to analyze the morphology characterization of FPN_AI_ using HT-7700 (Hitachi, Japan) at 200 kV. Zeta potentials and nanoparticle size were measured on Zetasizer Nano ZS90 (Malvern Instruments, UK) using dynamic light scattering (DLS) method. ^1^H-NMR analysis was conducted using Ascend 600 (Bruker, Swiss) to explore the molecular structure of the polymer chain. Fourier transform infrared spectroscopy (FTIR) analysis was conducted on Nicolet AVA TAR370 (ThermoFisher, USA) to identify functional groups of the polymer chain. Absorption spectrum was performed on Agilent Cary 6 (Marshall Scientific, USA) to measure the encapsulation efficiency, light transmittance and singlet oxygen generation of FPN_AI_. Fluorescence spectrum was carried out on Cary Eclipse (Agilent, USA) to determine the fluorescence intensity of FPN_AI_ at different pH values. The NIR light source was provided by an optical fiber-coupled 808 nm diode laser (STL808T1-7 W, Radium Laser Co., ltd, China). High performance liquid chromatography (HPLC) analysis was conducted on Agilent 1100 (Marshall Scientific, USA) to determine the concentration of ABT-263 and ICG in FPN_AI_.

### Fluorescence intensity measurement of FPN_AI_

The fluorescence intensity of FPN_AI_ solutions (0.1 mg/mL) was measured with a fluorescence spectrophotometer at 400 nm excitation and the solution pH value was adjusted from 7.4 to 6.0 by adding 0.1 M HCl.

### Singlet oxygen generation measurement of FPN_AI_

1,3-diphenylisobenzofuran (DPBF) dissolved in acetonitrile (20 µl, 8 mM) was mixed with FPN_AI_ solutions (0.1 mg/mL, 3 mL) at different pH values. The absorption spectra of mixed solutions were measured on a UV-vis spectrophotometer at the designed time intervals (0 min, 5 min, 10 min, 20 min, 25 min, and 30 min) after 808 nm laser (2.0 W/cm^2^) irradiation. All samples were stirred during being irradiated by the laser to ensure that the light energy was distributed evenly throughout the sample.

### *In vitro* drug release

Fresh PBS (5 mL) was added into an dialysis tube (MWCO: 3 kDa) (Spectra/Por, USA). The dialysis tube was then placed in another tube containing FPN_AI_ PBS solutions (0.1 mg/mL) with different pH values which was stirred at 100 rpm and 37℃. All PBS (5 mL) in the dialysis tube was removed and replaced with an equal amount of fresh PBS at the designated time intervals (0 h, 1 h, 3 h, 5 h, 7 h, 12 h, 24 h, 36 h, 48 h, 60 h, and 72 h). Finally, the amount of ABT-263 release were detected on a UV-vis spectrophotometer at 276 nm.

### Cell culture

Human HCC cell lines (Huh7, LM3, and HepG2), the mouse HCC cell line Hepa 1–6, and the normal hepatic epithelial cell line LO2 were purchased from FuHeng Biology (Shanghai, China). The normal mouse hepatocyte cell line AML12 was obtained from ATCC (CRL-2254). Cells were cultured with DMEM (Gibco, Carlsbad, CA, USA) or RPMI-1640 (Gibco, Carlsbad, CA, USA) supplemented with 10% fetal bovine serum (FBS) obtained from the Inner Mongolia Opcel Biotechnology Co., Ltd. (Inner Mongolia, China) and penicillin/streptomycin at 37 ℃ with 5% CO_2_.

### Cellular uptake of FNP_AI_ measured by immunofluorescence

LM3 (2 × 10^5^ /mL) and Huh7 (1.5 × 10^5^ /mL) cells were seeded into 6-well plates and cultured overnight. Then the medium was replaced to fresh medium with FPN_AI_ inside (1.0 µg/mL) and the pH values were set as 6.0 and 7.4. After incubating for 4 h, 12 h and 24 h, cells were treated with NIR. Next, cells were fixed using 4% paraformaldehyde for 10 min and stained with DAPI for 10 min subsequently. Finally, images were collected with fluorescent microscope.

To evaluate folate receptor-related targeting specificity, Folate Binding Protein (FBP) expression in LM3, Huh7, and AML12 cells was examined by western blot. Total protein was extracted, separated by SDS-PAGE, transferred onto PVDF membranes, and incubated with primary antibodies against FBP and β-tubulin.

For the competitive uptake assay, LM3, Huh7, and AML12 cells were seeded into culture plates and incubated overnight. Cells were pretreated with excess free folic acid for 1 h to block folate receptor binding sites, followed by incubation with PN_AI_ or FPN_AI_ under identical conditions. Cells without free folic acid preincubation were used as controls. After incubation, cells were washed, fixed with 4% paraformaldehyde, and stained with DAPI. Fluorescence images were acquired under identical imaging settings. The ICG fluorescence intensity was quantified and normalized to DAPI-positive cell area or cell number.

### Cellular uptake of FNP_AI_ measured by transmission electron microscope

LM3 (3 × 10^6^ /mL) and Huh7 (2.5 × 10^6^ /mL) cells were seeded into 10 cm-well plates and cultured overnight. Then the medium was replaced to fresh medium with FPN_AI_ inside (1.0 µg/mL) and the pH values were set as 6.0 and 7.4. After incubating for 4 h and 24 h, cell were treated with NIR. Then, cells were fixed with 2.5% glutaraldehyde (0.1 M, pH 7.0) overnight and post-fixed with 1% OsO4 for 1 h subsequently. Next, cells were dehydrated using a graded series of ethanol (30%, 50%, 70%, 80%) and acetone (90%, 95%, 100%) for 15 min. Then cells were incubated with a mixture of acetone and Spurr resin (1: 1 and 1: 3) for 1 h and 3 h, respectively, and incubated using Spurr resin overnight subsequently. After that, the samples were heated to 70 ℃ for 9 h, and were sectioned into slices by LEICA EM UC7. Finally, the sections were stained with uranyl acetate and alkaline lead citrate for 10 min, and observed by TEM at 120 kV (TecnaiG2 Spirit, Thermo Fisher Scientific, USA).

### Cell counting kit-8 (CCK-8) assay

Cell counting kit-8 (CCK-8) kit (Beyotime Biotechnology, Shanghai, China) was applied to measure the cytotoxicity of FPN_AI_ based on the manufacture’s protocol. Briefly, HCC and CCA cells were seeded into 24-well plates at a density of 3 × 10^4^/mL and incubated overnight. Then, FPN_AI_ was added and NIR irradiation was performed 24 h after that. Next, 100 µL CCK-8 reagent was added into each well and cells were incubated at 37 °C in dark for 1 h. Finally, absorbance at 450 nm wavelength was detected.

### Cell apoptosis assays measured by flow cytometry

Annexin V-FITC/PI cell apoptosis kit (Yeasen, Shanghai, China) was applied to measure the apoptosis of HCC cells based on the manufacture’s protocol. Briefly, HCC cells were seeded into 6-well plates at a density of 2 × 10^5^/mL and incubated overnight. Then, FPN_AI_ was added and NIR irradiation was performed 24 h after that. Next, cells were collected and re-suspended in a mixture of 1×binding buffer (100 µL), Annexin V-FITC staining solution (5 µL) and PI staining solution (10 µL). After incubating for 30 min, the apoptosis of cells was measured.

### Reactive oxygen species measurement

Reactive oxygen species (ROS) generated by HCC cells were detected by flow cytometry and immunofluorescence. LM3 (2 × 10^5^ /mL) and Huh7 (1.5 × 10^5^ /mL) cells were seeded into 6-well plates and cultured overnight. Then the medium was replaced to fresh medium with FPN_AI_ (1.0 µg/mL) and the pH values of the medium were set as 6.0 and 7.4. After incubating for 4 h, 12 h and 24 h, ROS dye was added and incubated for 30 min. Then, cell were treated with NIR. For flow cytometry, cells were collected and analyzed using flow cytometry (BD LSRFortessaTM, USA). For immunofluorescence, cells were fixed using 4% paraformaldehyde for 10 min and stained with DAPI for 10 min subsequently. Then, images were collected with fluorescent microscope.

### Western blot

Total protein was extracted from HCC cells by radioimmunoprecipitation assay (RIPA) buffer and quantified by bicinchoninic acid (BCA) Protein Assay Kit (Beyotime, Shanghai, China). Equal proteins were added into gels and electrophoresed for 30 min at 200 V flowed by 50 min at 80 V to separate the proteins. Then, all proteins were transferred to polyvinylidene difluoride (PVDF) membranes through 350 mA for 70 min. After that, membranes were blocked using milk for 1 h and incubated with primary antibodies at 4 °C overnight. Secondary antibodies were then employed to incubate the membranes for 1 h at room temperature. Protein bands were visualized through the enhanced chemiluminescence (ECL) detection kit (ThermoFisher, Waltham, MA, USA). The information for antibodies were: anti-BCL2 (Cat: 68142), anti-BCL-xL (Cat: 63370), anti-Mcl-1 (Cat: 50480), anti-Caspase (Cat: 65673) and anti-GAPDH (Cat: 61924). All antibodies were purchased from GenuIN biotech (Hefei, China) and the dilution were 1: 1000.

### Animal model and *in vivo* treatment

Two types of mice model were applied, which were constructed on male BALB/c nude mice (4 weeks old) and c57BL/6 mice (4 weeks old) purchased from GemPharmatech (Nanjing, China). First, 5 × 10^6^ Huh7 cells in 200 µL PBS were subcutaneously injected into the left armpit of each BALB/c nude mice. When the size of tumors reached 200 mm^3^, tumors were collected and cut into small lumps with a diameter of 1 cm. Then, these small tumors were transplanted into the left armpit of mice. Second, 5 × 10^6^ Hepa 1–6 cells in 200 µL PBS were subcutaneously injected into the right armpit of each c57BL/6 mice.

Treatments were given when the tumor volume reached 150–200 mm^3^, which is calculated as π/6 × (tumor length) × (tumor width) × (tumor height). For treatments, mice were divided randomly into 4 groups with 5 mice in each group: (1) PBS, (2) ABT-263 (equivalent to the dose loaded in FPN_AI_), (3) FPN_AI_, and (4) FPN_AI_ with laser irradiation (FPN_AI_ + NIR, the amount of ABT-263 and ICG were 4 mg/kg and 5 mg/kg, respectively).

All formulations were administered intravenously via the tail vein. To further evaluate whether the therapeutic effect of FPN_AI_ resulted from nanoparticle-mediated co-delivery rather than simple co-administration of free drugs, additional free drug solution mixture control groups were included in the animal experiment, including free ABT-263 plus free ICG and free ABT-263 plus free ICG with NIR irradiation. The doses of free ABT-263 and free ICG were equivalent to those loaded in FPN_AI_, and the NIR irradiation protocol was identical to that used for the FPN_AI_ + NIR group. In this additional formulation-comparison experiment, each group included four mice.

The in vivo doses and drug ratio were selected based on the measured loading contents of FPN_AI_, formulation stability, previously reported effective dose ranges of navitoclax- and ICG-related therapeutic systems, and tolerability considerations for systemic administration. The ABT-263 dose was kept equivalent between the free ABT-263 and FPN_AI_ groups to allow direct comparison of formulation-dependent therapeutic efficacy. A previous navitoclax-containing nanoparticle study used a 4 mg/kg intravenous dose and achieved marked antitumor efficacy without overt toxicity, supporting the feasibility of this dose range [17]. The ICG dose was selected to provide sufficient NIR responsiveness, and an intravenous dose of 5 mg/kg ICG has been used in preclinical tumor imaging and NIR-mediated phototherapy models, including HCC xenograft models [26]. Therefore, the ABT-263/ICG ratio used in this study was selected as a feasibility-oriented regimen for evaluating FPN_AI_-mediated combination therapy rather than as a fully optimized dose combination.

The power density and time of irradiation were 2.5 W/cm^2^ for 10 min, with a 1 min interval after each minute of irradiation to prevent tissue heating. Tumor volumes were measured every three days, and the mice were euthanized after six treatment cycles. Blood samples from the PBS and FPN_AI_ groups were collected for routine blood tests and liver function assays. The heart, liver, spleen, lung, and kidney were harvested from the PBS and FPN_AI_ groups for H&E staining to assess the biosafety of FPN_AI_.

### Immunofluorescence (IF)

The slides were treated with 0.1% Triton X-100 for 20 min to permeate and with 5% bovine serum albumin for 40 min to block subsequently. Then, primary antibodies were incubated overnight at 4 ℃ and the secondary antibody was hired to incubate for 1 h at room temperature. Finally, DAPI was employed to incubate for 10 min and photos were obtained. The information for antibodies were: anti-calreticulin (Cat: T55353), anti-high mobility group box 1 (Cat: T55060), anti-CD4 (Cat: TB7039), anti-CD8 (Cat: PC0648), anti-CD11c (Cat: TD7585), anti-CD80 (Cat: PC23738), anti-granzyme B (Cat: PC1351) and anti-perforin (Cat: PC7128). All antibodies were purchased from Abmart Shanghai Co.,Ltd. (Shanghai, China) and the dilution were 1: 100.

### H&E staining

The samples were fixed by 4% paraformaldehyde overnight and slides were prepared. The slides were baked in an incubator (60℃) and for 3 h. Then, the slices were dewaxed and hydrated using xylene (10 min, twice), absolute ethanol (5 min, once), 95% ethanol (5 min, once), 85% ethanol (5 min, once), and 75% ethanol (5 min, once) orderly. After that, hematoxylin dye solution (2 min), 1% hydrochloric acid and alcohol dropwise (2–30 s), and eosin staining solution (30–120 s) were used in turn. Next, the slides were dehydrated using 70% ethanol (10 s), 85% ethanol (10 s), 95% ethanol, and absolute ethanol (10 s) in turn, and been transparent using xylene for 5 min. Finally, the slides were scanned and photos were collected.

### Blood test

Complete blood investigation and liver function experiments were conducted after using blood obtained from the mice. For complete blood investigation, the white blood cells (WBC), platelets (PLT), red blood cells (RBC), mean corpuscular volume (MCV), and hemoglobin concentration (MCHC) were detected. For liver function assay, alanine aminotransferase (ALT), aspartate aminotransferase (AST), total protein (TP), albumin (ALB), and direct bilirubin (DBIL) were measured.

### Hemolysis assay

To evaluate the hemocompatibility of FPN_AI_ for intravenous administration, an in vitro hemolysis assay was performed. Fresh anticoagulated mouse blood was centrifuged, and red blood cells were collected and washed with PBS until the supernatant became clear. The red blood cell suspension was incubated with PBS as the negative control, Triton X-100 as the positive control, or FPN_AI_ at 1×, 2×, and 5× concentrations at 37 °C. After incubation, the samples were centrifuged, and the supernatants were photographed and measured at 540 nm to quantify hemoglobin release. The hemolysis rate was calculated using the following equation: hemolysis rate (%) = [(A_sample − A_PBS)/(A_Triton − A_PBS)] × 100%. All experiments were performed with five replicates.

### *In vivo* imaging

Free ICG and FPN_AI_ were intravenously injected separately into two mice bearing HCC tumor constructed as above. Fluorescence images of these mice were obtained through the NIR imaging system (PerkinElmer IVIS^®^ Lumina LT, USA) at 0 h, 0.5 h, 1 h, 2 h, 3 h, 6 h, and 12 h postinjection.

### Statistical analysis

All data were shown as means and standard errors. Graphpad Prism 9.4.1 (GraphPad Software, La Jolla, CA, USA) was hired for the Student’s t-test, Wilcoxon matched-pairs test, Fisher exact test and chi-square test. Kaplan-Meier analysis was employed for survival analysis. *P* value < 0.05 were regarded as statistically significant.

## Results

### Synthesis and characterization of FPN_AI_

The polymer chain of FPN_AI_ is primarily composed of PEG hydrophilic group and polyaspartic acid hydrophobic group. To synthesize the hydrophobic group, BLA-NCA was first prepared using triphosgene (Figure S1a). Subsequently, the amino group in PEG was utilized as an initiator to induce ring-opening polymerization of BLA-NCA, which connected the hydrophilic and hydrophobic groups and ultimately formed the template chain (Figure S1b). Due to the high expression of FR on the membrane of HCC cells (Figure S2), packing FA further enhances the HCC-specific targeting capability of FPN_AI_ (Figure S1c). To provide pH sensitivity, the API group was introduced into the branched chain via an aminolysis reaction (Figure S1d). The successful synthesis of polymer chain in FPN_AI_ was confirmed via ^1^H-NMR. The peaks at δ = 7.4 and 5.1 ppm were attributed to the protons of hydrophobic group, and the peaks at δ = 3.5 ppm were attributed to the protons of PEG (Figure S3, S4). Additionally, signals at δ = 8.4, 7.7, 6.7, 4.5 and 4.3 ppm were attributed to the protons of FA (Figure S5). Finally, the new peaks at δ = 9.2, 7.8 and 7.7 ppm were attributed to the protons of the API’s imidazole group (Figure S6). All these characteristic peaks meet the standards, indicating the successful synthesis of PEG-p(API-ASP)-FA.

**Fig. 1:**
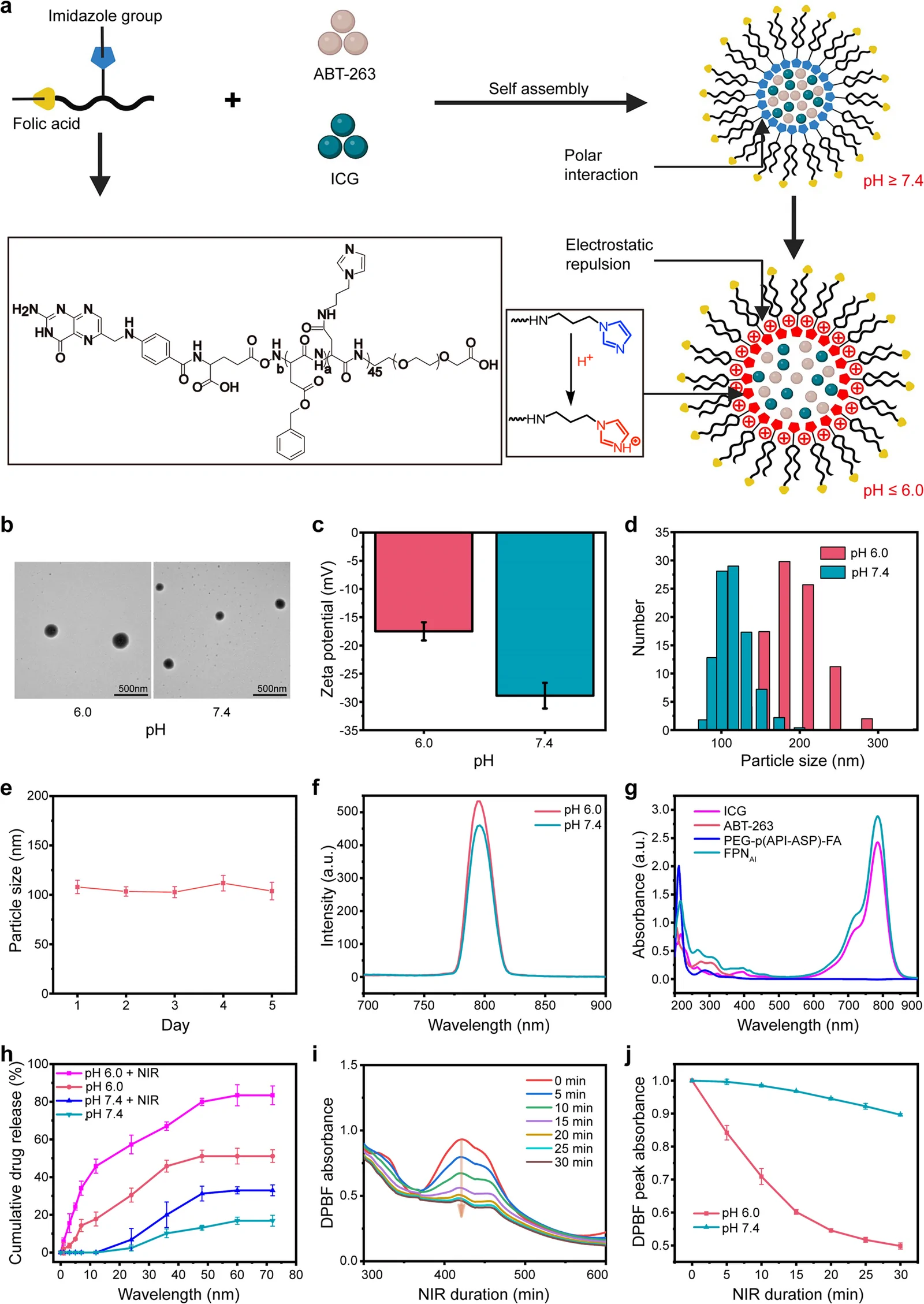
Characterization of FPN_AI_. (**a**) Schematic diagram of tumor pH activation and NIR irradiation of FPN_AI_. (**b**) TEM images of FPN_AI_ at pH 7.4 and pH 6.0 after NIR irradiation. (**c**) Zeta potential changes of FPN_AI_ at pH 7.4 and pH 6.0. (**d**) FPN_AI_ particle size distribution at pH 7.4 and pH 6.0. (**e**) Stability test of FPN_AI_ in PBS via DLS measurements. (**f**) ICG fluorescence intensity from FPN_AI_ at pH 7.4 and pH 6.0. (**g**) Fluorescence intensity of ICG, ABT-263, PEG-p(API-ASP)-FA, and FPN_AI_. (**h**) In vitro drug release profile of FPN_AI_. (**i**) FPN_AI_ DPBF absorbance spectrum changes at pH 6.0 under NIR irradiation over multiple time intervals. (**j**) FPN_AI_ pH-dependent DPBF absorbance under NIR irradiation at pH 7.4 and pH 6.0. The data are represented as mean ± SD (*n* = 3)

To fabricate FPN_AI_, ABT-263 and ICG were loaded into the hydrophobic core via a self-assembly approach. The FPN_AI_ is pH-responsive, and the imidazole group in FPN_AI_ activates and ionizes as the pH decreases, causing swelling and instability of the nano drug delivery system. This ensures targeted drug delivery in the acidic tumor microenvironment (TME), and avoids systemic side effects (Fig. 1a). Additionally, the loading of ICG offers photoactivity, providing a second layer of protection to precision cancer therapy. To evaluate the pH-responsiveness of FPN_AI_, transmission electron microscopy (TEM) is commonly employed to characterize the NP microstructures. As shown in Fig. 1b, at pH = 7.4 and 6.0, the distribution of FPN_AI_ is uniform and spherical, but the spherical volume is larger at pH = 6.0, suggesting that FPN_AI_ is swollen. Then, the zeta potential and particle size of FPN_AI_ at different pH values were detected. The zeta potential of FPN_AI_ is -28.9 mV and the average hydrodynamic diameter is about 120 nm at pH = 7.4. When exposed to an acidic microenvironment, the surface negative charge reduced to -17.5mV because of the protonation of imidazole group, and the average hydrodynamic diameter increased to about 200 nm due to the osmotic swelling property of FPN_AI_ (Fig. 1c and d). Further, the size of FPN_AI_ was measured by DLS method for five consecutive days after placing it in PBS, and we found that the size of FPN_AI_ keep stable in the five days (Fig. 1e). Above data revealed that the synthesis of FPN_AI_ NPs is successful and its structural stability is high.

Under physiological conditions, the photoactivity and fluorescence intensity of ICG in FPN_AI_ are significantly quenched due to mutual aggregation. As the pH decreases, FPN_AI_ gradually swells, reducing structural stability and recovering both ICG photoactivity and fluorescence intensity (Fig. 1f). To further characterize FPN_AI_, UV-visible absorption spectrum was conducted. According to the results, the peak at 306 nm corresponds to ABT-263, and the peak at 784 nm corresponds to ICG. Both were detected simultaneously in UV-visible absorption spectrum of FPN_AI_, confirming its successful fabrication (Fig. 1g). Based on the HPLC results, the loading efficiencies of ABT-263 and ICG in FPN_AI_ were approximately 14.8% and 11.1%, respectively (Figure S7).

To investigate the potential biomedical applications of FPN_AI_, we used the reverse dialysis method to perform in vitro drug release of FPN_AI_ (Fig. 1h). The results showed that about 17% and 51% ABT-263 were released from FPN_AI_ after 72 h under pH = 7.4 and 6.0 respectively, indicating the excellent pH-responsive characteristics of FPN_AI_. Additionally, about 17% and 33% ABT-263 were released from FPN_AI_ after 72 h under pH = 7.4 without or with irradiation, demonstrating that the excellent stability of FPN_AI_ under physiological conditions can prevent the release of ABT-263 under NIR irradiation. Besides, the release rate of ABT-263 after NIR irradiation is as high as 83.4% at pH = 6.0, proving the outstanding NIR response characteristics of FPN_AI_. Generally speaking, these results showed that FPN_AI_ is a kind of NPs with excellent pH and NIR response activity. Next, to investigate the pH-dependent generation of ROS from FPN_AI_, we then measured the generation of ^1^O_2_ by using DPBF as an indicator because ^1^O_2_ can irreversibly quench the ultraviolet characteristic absorption peak of DPBF. Only a slight decrease of the absorption intensity of DPBF was detected with time after NIR irradiation at pH = 7.4, while a dramatic drop of that was found at pH = 6.0 (Fig. 1i, Figure S8). Finally, to display the difference of ^1^O_2_ generation at pH = 7.4 and 6.0 more clear, we plotted a figure showing the relative DBPF peak absorption with time at the two pH value, which also exhibited the superiority of ^1^O_2_ production in the case of pH = 6.0 (Fig. 1j). In conclusion, we have successfully synthesized FPN_AI_, and the features of FPN_AI_ meet the standard.

### Cellular uptake and cytotoxicity of FPN_AI_*in vitro*

**Fig. 2:**
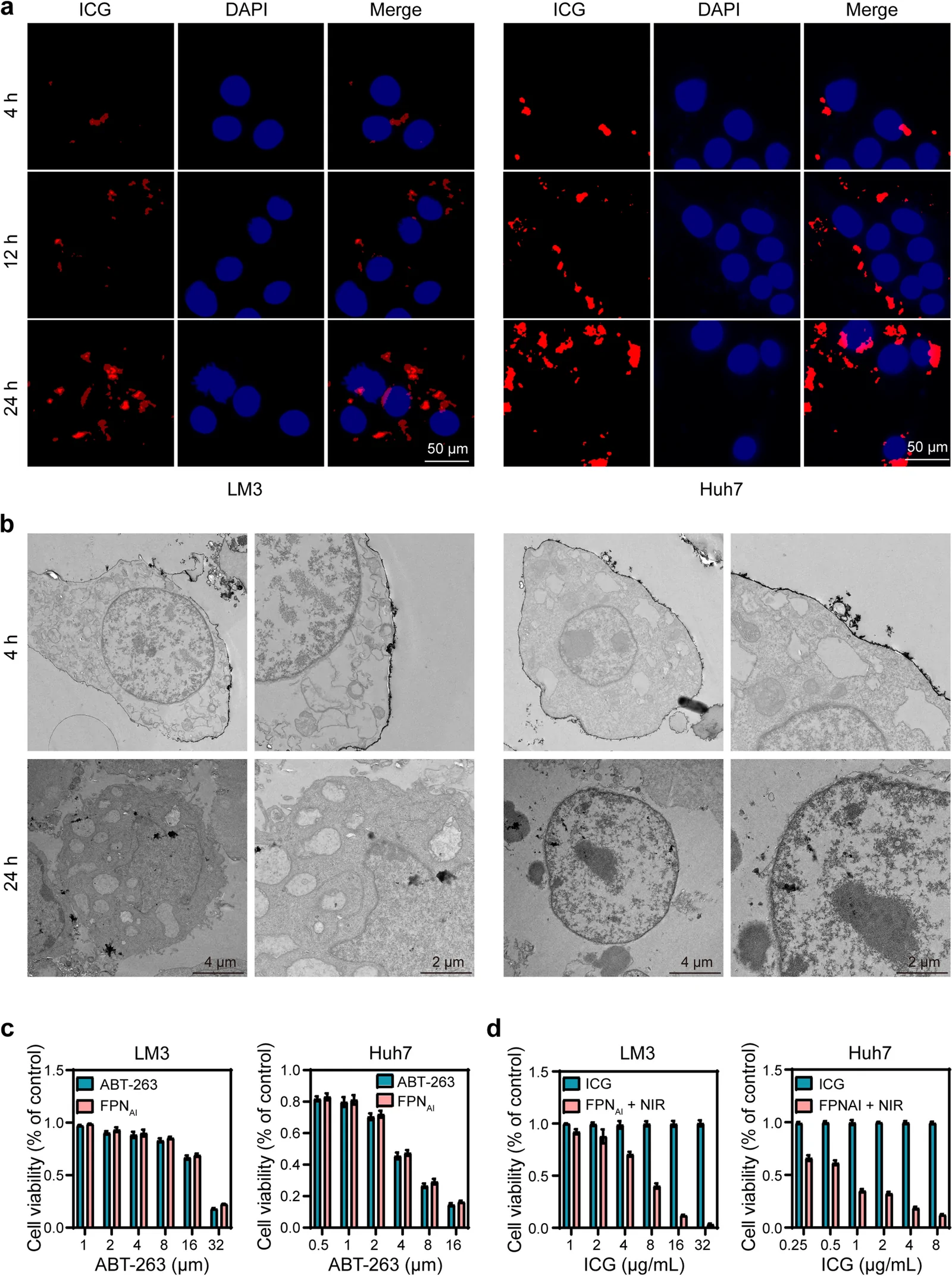
FPN_AI_ interactions with HCC cells. (**a**) Immunofluorescence images of FPN_AI_ cellular uptake by HCC cells at different time points at pH = 6.0. FPN_AI_ fluorescence signals are exhibited in red and DAPI stained cells are displayed in blue. (**b**) TEM images of FPN_AI_ cellular uptake by HCC cells at different time points at pH 6.0. (**c**) CCK-8 assays of HCC cells treated with ABT-263 at different concentrations (equivalent to the dose in FPN_AI_. **d**) 48 h CCK-8 assays of HCC cells exposed to different concentrations of ICG with 808 nm laser irradiation. The data are represented as mean ± SD (*n* = 3)

The cellular uptake of FPN_AI_ was observed by IF and TEM. As displayed in Fig. 2a, FPN_AI_ was gradually absorbed by cells over time, and intracellular FPN_AI_ was mainly distributed around the nucleus of cells. Similarly, the images obtained from TEM also revealed that FPN_AI_ can be quickly endocytosed into the cells and converge surround the nucleus of HCC cells **(**Fig. 2b**)**. Above data indicate that FPN_AI_ can be effectively endocytosed by tumor cells in acidic TME.

To verify whether the folate modification contributed to tumor cell targeting, we further examined FBP expression and performed a competitive uptake assay. Western blot analysis showed that LM3 and Huh7 cells expressed higher levels of FBP than AML12 cells (Figure S11 a, b). We then compared the uptake of non-folate-modified PN_AI_ and folate-modified FPN_AI_ with or without free FA preincubation. In LM3 and Huh7 cells, FPN_AI_ showed stronger fluorescence signals than PN_AI_, whereas free FA preincubation markedly attenuated FPN_AI_ uptake. In contrast, free FA had no obvious effect on PN_AI_ uptake. AML12 cells showed weak overall fluorescence signals and no evident folate-dependent enhancement (Figure S11 c–f). These findings indicate that FPN_AI_ uptake in HCC cells is at least partly associated with folate receptor-mediated targeting.

Moreover, the cytotoxicity of FPN_AI_ and the enclosed ICG under NIR irradiation was explored via CCK-8 assay. No additional cytotoxicity to tumor cells was caused by FPN_AI_ compared with ABT-263 at an equal concentration, and the cell-killing effect of FPN_AI_ was slightly weaker than free ABT-263 because of the slow and incomplete release rate of ABT-263 from FPN_AI_ without NIR irradiation (Fig. 2c). Additionally, single encapsulated ICG is harmless to HCC cells within a certain concentration range, but obvious cell-killing effect can be detected after receiving NIR irradiation [NIR intensity is 808 nm (2 W/cm^2^) and time of irradiation is 2 min] **(**Fig. 2d**)**. Thus, FPN_AI_ can be absorbed by HCC cells.

### *In vitro* anti-tumor ability of FPN_AI_

**Fig. 3:**
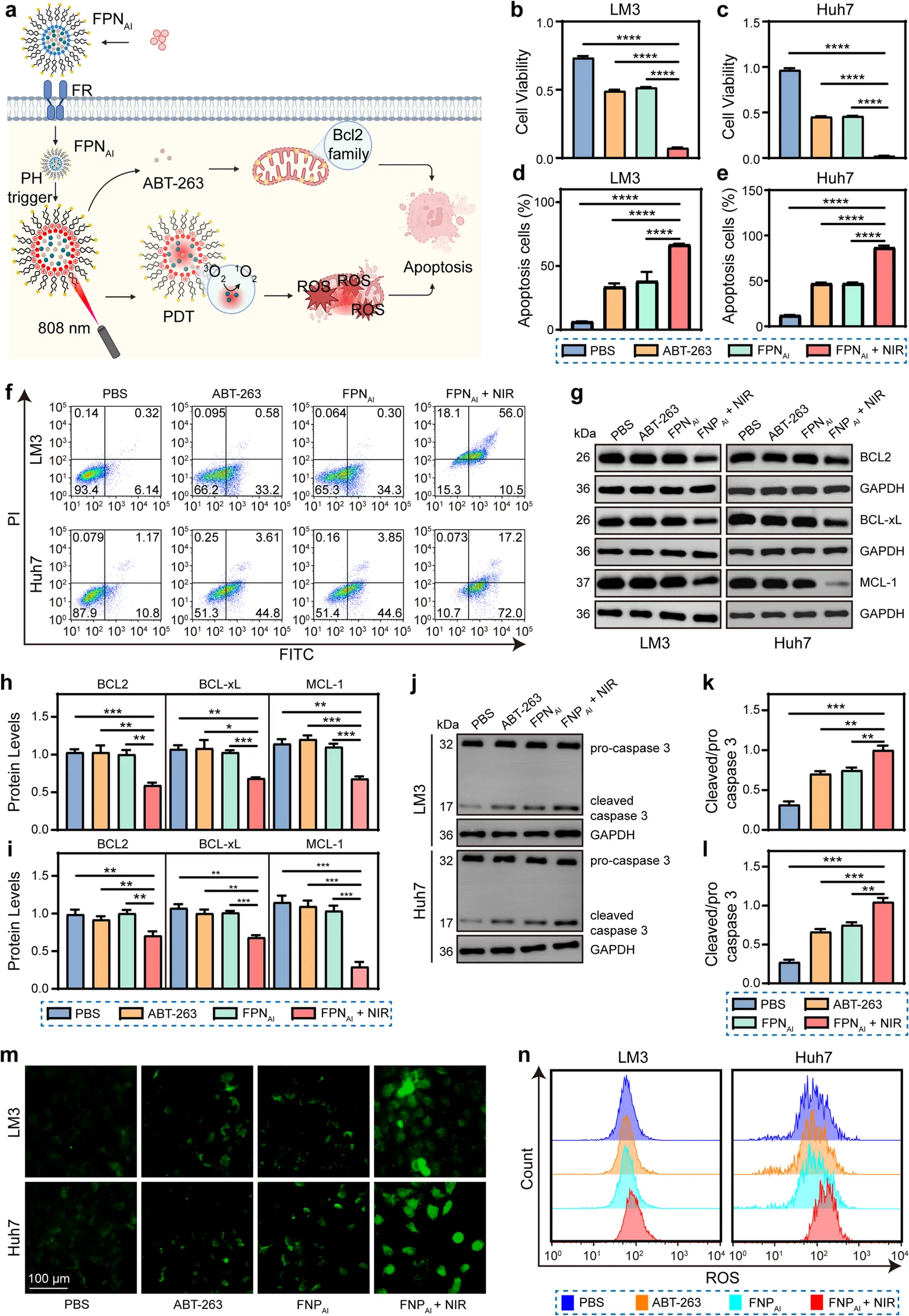
In vitro antitumor activity of FPN_AI_. **a**) Schematic illustration of the molecular mechanism underlying the effects of FPN_AI;_ **b-c**) CCK-8 assays of HCC cells; **d-f)** flow apoptosis experiment of HCC cells; **g-i**) Levels of Bcl-2, BCL-xL and Mcl-1 in HCC cells measured by western blot; **j-l**) Levels of pro- and cleaved- caspase 3 in HCC cells measured by western blot; **m-n**) ROS levels in HCC cells measured by immunofluorescence. The intensity and time of irradiation in this part were 2 W/cm^2^ for 5 min with every min interval. The data are represented as mean ± SD (*n* = 3). ***P < 0.001

According to the features of FPN_AI_, it was expected to induce apoptosis of HCC cells (Fig. 3a). To verify this hypothesis, CCK-8 and flow cytometry assays were performed. Cells were divided into PBS group, ABT-263 group, FPN_AI_ group and FPN_AI_ + NIR group. The results of CCK-8 assay showed that the survival rates of HCC cells in FPN_AI_ group were slightly higher than those in ABT-263 group at an equal concentration, suggesting the excellent controlled release capacity of the carriers. Specially, the survival rates of HCC cells dropped dramatically in FPN_AI_ + NIR group, indicating the predominant synergistic anti-proliferative effect of ABT-263 and ICG based photodynamic therapy (PDT) (Fig. 3b-c). Likewise, the data of flow cytometry assay also demonstrated the obvious effect of FPN_AI_ + NIR in inducing apoptosis of HCC cells (Fig. 3d-f, Figure S5). Above results indicate that the efficacy of FPN_AI_ + NIR is better than that of ABT-263 or ICG-based PDT.

### FPN_AI_ induced mitochondria associated apoptosis and ROS burst

ICG with NIR irradiation can induce electrons to rise from the ground state to singlet and triplet states, thus producing numerous ROS, which in turn kills cancer cells [27, 28]. Also, ICG with NIR irradiation can induce the down-regulation of pro-survival proteins Bcl-2, Bcl-xl and Mcl-1 [29]. ABT-263 can activate caspase 3 through affecting the combination of Bcl-2/Bcl-xl with pro-apoptotic proteins Bax and Bak, thus inducing apoptosis of tumor cells. However, long-term application of ABT-263 may cause increased expression of pro-survival protein Mcl-1 and ABT-263 can not decrease the levels of Bcl-2/Bcl-xl, which limit its application. FPN_AI_ contains ABT-263 and ICG, so theoretically, FPN_AI_ + NIR can induce apoptosis mediated by mitochondria and ROS. To verify this hypothesis, we first detected the levels of Bcl-2, Bcl-xl, Mcl-1and cleaved caspase 3 in HCC cells using western blot. Interestingly, the levels of Bcl-2, Bcl-xl and Mcl-1 decreased and the ratio of cleaved-caspase 3/pro-caspase 3 increased in FPN_AI_ + NIR groups (Fig. 3g-l). Also, we detected the generation of ROS in HCC cells with different treatments using immunofluorescence and flow cytometry. The results of both experiments showed that the generation of ROS increases significantly in HCC cells in FPN_AI_ + NIR group (Fig. 3m-n). Overall, FPN_AI_ with NIR irradiation can induce both mitochondria and ROS mediated cell apoptosis.

### *In* vivo anti-tumor effect of FPN_AI_

**Fig. 4:**
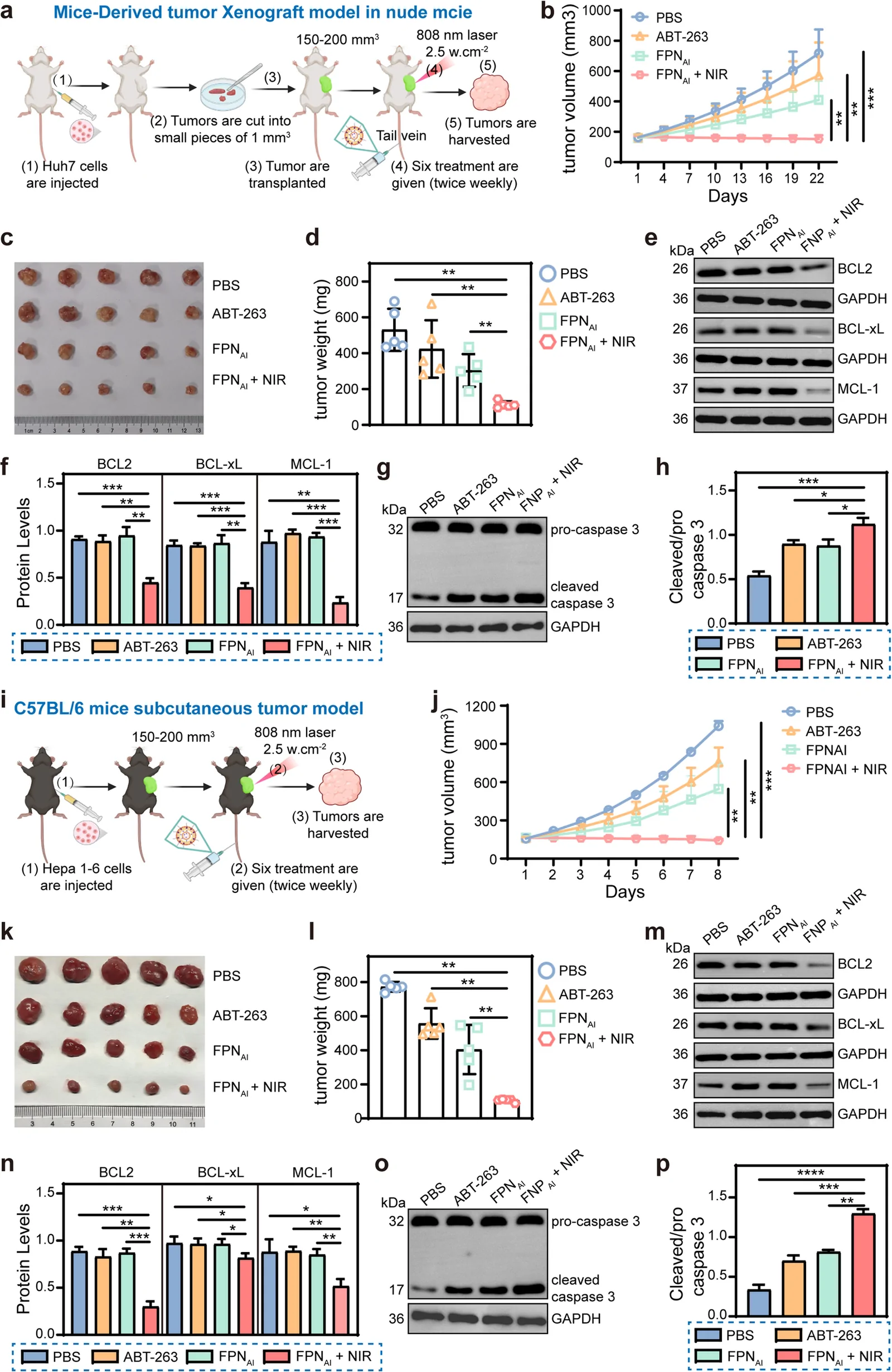
In vivo antitumor therapeutic effect of FPN_AI_. (**a**) Schematic illustration of CDX animal model construction and treatment. (**b**) Changes of tumor volume in nude mice over time. (**c**) The average tumor weights in nude mice at day 21. (**d**) Tumor gross images in nude mice at day 21. (**e-f**) Levels of Bcl-2, BCL-xL and Mcl-1 in HCC tissues measured by western blot. (**g-h**) Levels of pro- and cleaved- caspase 3 in HCC tissues measured by western blot. (**i**) Schematic illustration of C57BL/6 mice subcutaneous tumor model construction and treatment. (**j**) Changes of tumor volume in C57BL/6 mice over time. (**k**) The average tumor weights in C57BL/6 mice at day 8. (**l**) Tumor gross images in C57BL/6 mice at day 8. (**m-n**) Levels of Bcl-2, BCL-xL and Mcl-1 in HCC tissues measured by western blot. (**o-p**) Levels of pro- and cleaved- caspase 3 in HCC tissues measured by western blot. There are five mice in each group. The data are represented as mean ± SD (*n* = 5 mice per group). **P < 0.01, ***P < 0.001

Huh7 cell-derived xenograft HCC models were employed to analyze the anti-tumor effect of our NPs. When the tumor volume reached 150–200 mm^3^, treatments were given (Fig. 4a). Compared with PBS, all the three treatments can result in tumor growth inhibition. Notably, FPN_AI_ + NIR showed the best anti-tumor efficacy throughout the experimental period, which was substantially superior to that of ABT-263 and FPN_AI_ (Fig. 4b). After euthanizing the mice after 6 procedures, the tumors were collected and then photographed and weighed, with similar trend as tumor volume monitored continuously (Fig. 4c-d). Besides, the levels of Bcl-2, Bcl-xl and Mcl-1 decreased and the ratio of cleaved-caspase 3/pro-caspase 3 increased in FPN_AI_ + NIR groups (Fig. 4e-h).

Hepa 1–6 cells were employed to construct a subcutaneous tumor model in C57BL/6 mice (Fig. 4i). Similarly, FPN_AI_ + NIR showed the best anti-tumor efficacy (Fig. 4j-l). Also, the levels of targeted proteins exhibited similar trend in C57BL/6 mice (Fig. 4m-p). Above data confirmed that FPN_AI_ + NIR show the best anti-tumor capacity compared with other methods and FPN_AI_ + NIR can overcome the limitation of ABT-263 on regulation Bcl-2 family.

To further determine whether FPN_AI_+NIR outperformed simple co-administration of the two free agents, we added free ABT-263 plus free ICG mixture groups with or without NIR irradiation. In both Huh7 xenograft and Hepa1-6 tumor models, the free drug mixture groups showed moderate tumor growth inhibition compared with PBS. However, FPN_AI_ + NIR consistently achieved stronger tumor suppression than the free drug mixture groups, as shown by tumor growth curves, endpoint tumor weights, and gross tumor images (Figure S12). These results further support the therapeutic advantage of nanoparticle-mediated co-delivery and NIR-responsive activation over simple free-drug co-administration.

### The effect of FPN_AI_ on ICD

**Fig. 5:**
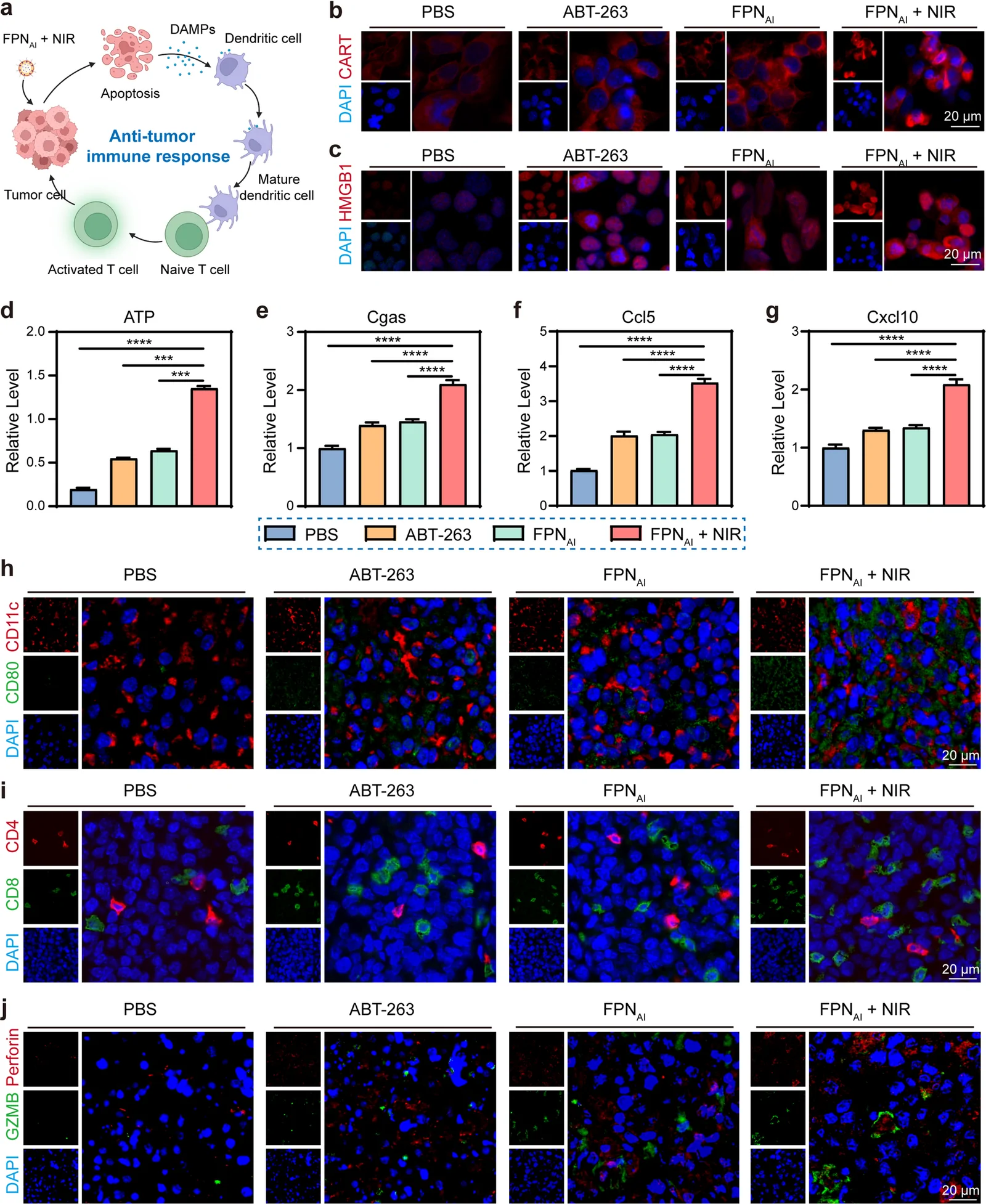
The Effect of FPN_AI_ on ICD. (**a**) Schematic illustration of ICD; (**b-c**) IF staining of CART and HMGB1 in HCC cells; (**d-g**) The levels of ATP, Cgas, Ccl5 and Cxcl10 in HCC cells; (**h**) IF staining of CD11c and CD80 in HCC tissues; (**i**) IF staining of CD4 and CD8 in HCC tissues; (**j**) IF staining of GZMB and perforin in HCC tissues. The data are represented as mean ± SD (*n* = 5 mice per group). **P < 0.01, ***P < 0.001

ICD is defined by the release of a panel of DAMPs from dying cells, such as surface exposed calreticulin (CART) and high mobility histone 1 (HMGB1). These DAMPs are efficiently recognized and engulfed by DCs, inducing DCs maturation, facilitating T cells activation, and ultimately initiating a potent anti-tumor immune response to eradicate tumor cells. Because FPN_AI_ can induce both mitochondria-related and ROS-induced HCC cells apoptosis, we hypothesized that FPN_AI_ can also trigger ICD (Fig. 5a). The in vitro IF indicated that there exist much more abundant CART and HMGB1 in HCC cells after treatment, especially in FPN_AI_ group (Fig. 5b-c). Additionally, levels of other DAMPs, including ATP, Cgas, Ccl5 and Cxcl10, also confirmed the effect of FPN_AI_ in inducing ICD (Fig. 5d-g). Further, in vivo IF staining CD11c (a marker for DCs) and CD80 (a marker for mature DCs) demonstrated the increased percentage of mature DCs in FPN_AI_ group (Fig. 5h). Also, in vivo IF staining of CD4 and CD8 illustrated the enhanced differentiation of T cells toward CD8^+^ T cells in FPN_AI_ group (Fig. 5i). In addition, in vivo IF staining of factors secreted by CD8^+^ T cells, granzyme B (GZMB) and perforin, confirmed the elevation of CD8^+^ T cells in FPN_AI_ group (Fig. 5j). Overall, HCC cells tend to release DAMPs and trigger ICD following FPN_AI_ treatment.

### Tumor targeting ability and biological safety of FPN_AI_

**Fig. 6:**
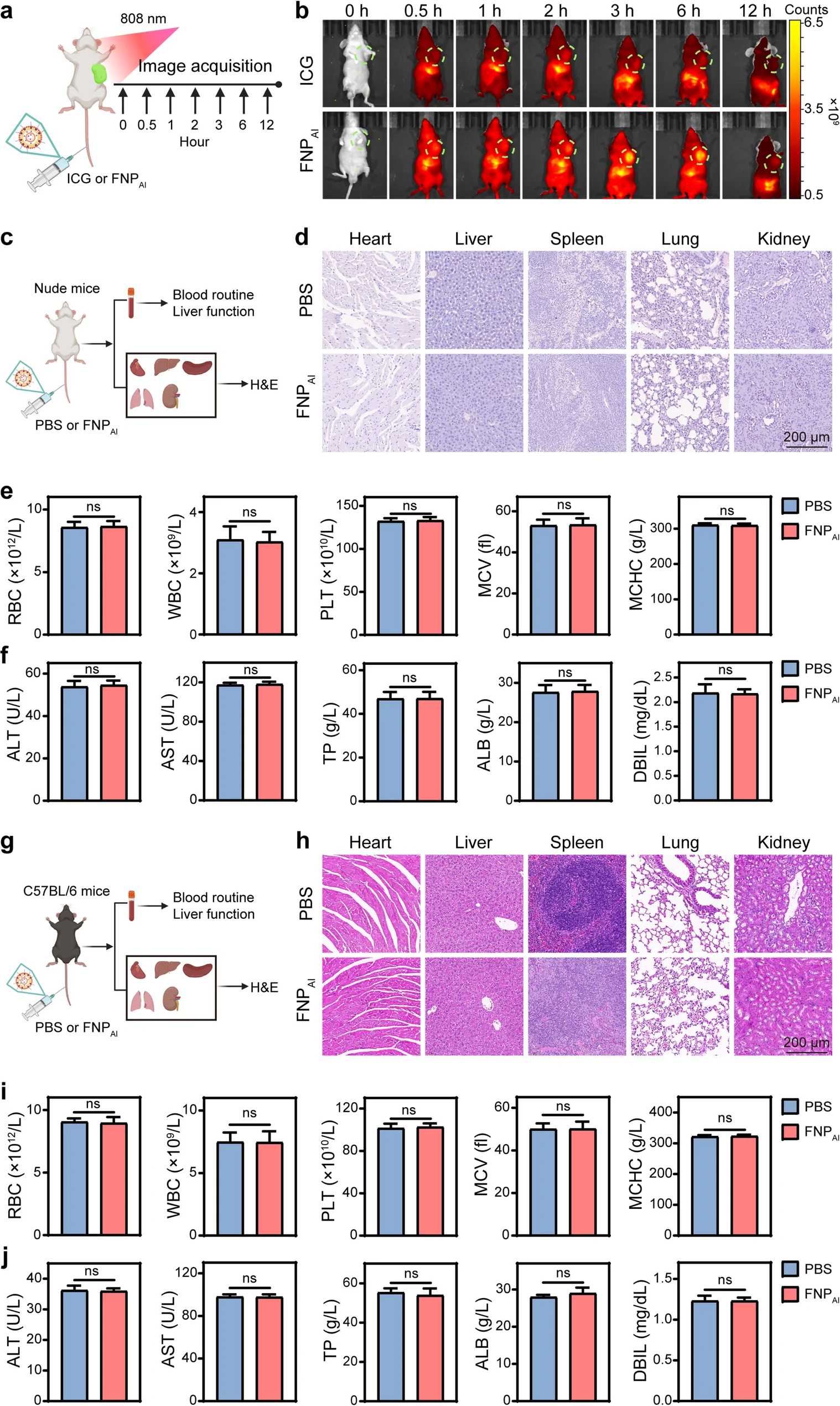
Tumor-targeting ability and bio-safety of FPN_AI_. (**a**) Schematic illustration of in vivo imaging; (**b**) In vivo imaging of mice at 0 h, 0.5 h, 1 h, 2 h, 3 h, 6 h and 12 h post intravenously injection of ICG and FPN_AI_; (**c**) Schematic illustration of detecting bio-safety in nude mice; (**d**) Representative H&E staining of heart, liver, spleen, lung an kidney in nude mice; (**e**) results of RBC, WBC, PLT, MCV and MCHC in the blood of nude mice; (**f**) results of ALT, AST, TP, ALB and DBIL in the blood of nude mice; (**g**) Schematic illustration of detecting bio-safety in C57BL/6 mice; (**h**) Representative H&E staining of heart, liver, spleen, lung an kidney in C57BL/6 mice; (**i**) results of RBC, WBC, PLT, MCV and MCHC in the blood of C57BL/6 mice; (**j**) results of ALT, AST, TP, ALB and DBIL in the blood of C57BL/6 mice. There are five mice in each group. The data are represented as mean ± SD, *n* = 5 mice per group

ICG or FPN_AI_ was injected intravenously into HCC bearing nude mice at an equivalent dose of ICG, and in vivo imaging was performed to evaluate the tumor targeting capacity of FPN_AI_ at different time points (Fig. 6a). As shown in Fig. 6b, tumor regions were indicated with green dashed circles, suggesting the excellent tumor targeting ability of FPN_AI_. ROI-based quantification of tumor fluorescence further showed that FPN_AI_ generated stronger and more sustained tumor-region fluorescence than free ICG over the observation period (Figure S13). These results indicate that FPN_AI_ exhibits enhanced tumor accumulation and prolonged retention compared with free ICG.

Bio-safety of FPN_AI_ was also assessed in both nude mice and C57BL/6 mice. In nude mice, blood and main organs were collected from both FPN_AI_ and PBS groups (Fig. 6c). First, routine blood tests and liver function tests were measured to assess the systemic toxicology of FPN_AI_, which exhibited no statistically significant differences (Fig. 6d and e). Meanwhile, H&E staining of heart, liver, spleen, lung and kidney revealed no obvious toxicology of FPN_AI_ (Fig. 6f). In C57BL/6 mice, blood and main organs were also harvested (Fig. 6g). The results of routine blood test, liver function test and H&E staining also indicated the bio-safety of FPN_AI_ (Fig. 6h-j).

Considering the intravenous administration route of FPN_AI_, we further evaluated its hemocompatibility using an in vitro hemolysis assay. Red blood cells were incubated with PBS, Triton X-100, or FPN_AI_ at 1×, 2×, and 5× concentrations. The supernatants of the FPN_AI_-treated groups remained similar to that of the PBS group, whereas Triton X-100 caused obvious hemoglobin release. Consistently, OD540-based quantification showed that the hemolysis rates of FPN_AI_ remained below 5% even at the 5× concentration (Figure S14). These results indicate that FPN_AI_ has favorable blood compatibility for intravenous administration.

Collectively, above data confirmed the excellent tumor targeting ability and favorable biological safety of FPN_AI_ in vivo.

## Discussion

The development of the folic acid–modified, dual pH- and near-infrared (NIR)–responsive nanoparticle FPN_AI_ addresses key challenges in hepatocellular carcinoma (HCC) therapy by coupling targeted delivery with multi-modal anti-tumor mechanisms. Stimuli-responsive nanocarriers have been widely explored to enhance tumor specificity and reduce systemic toxicity by exploiting the acidic tumor microenvironment (TME) and other pathophysiological cues [30, 31]. Recent nanomedicine studies have further emphasized that tumor-responsive materials should not only improve drug delivery, but also remodel the tumor microenvironment and integrate multiple therapeutic cues, such as redox modulation, immune activation, and microenvironment-adaptive release [32, 33]. The ability of FPN_AI_ to maintain structural stability under physiological pH and undergo controlled swelling in acidic conditions aligns with established principles of pH-triggered drug release, which have been shown to improve intratumoral drug accumulation and therapeutic index [34]. Compared with conventional pH-responsive nanocarriers that mainly function as stimulus-triggered delivery vehicles, FPN_AI_ integrates multiple therapeutic modules within a single formulation. The folic acid ligand provides folate receptor-associated tumor cell targeting, the imidazole-containing polymer enables acidic pH-responsive swelling and release, and the co-loaded ABT-263/ICG combination links BH3 mimetic-induced mitochondrial apoptosis with NIR-activated ROS generation. This integrated design allows FPN_AI_ to function not only as a pH-responsive carrier but also as a combined apoptosis- and photodynamic therapy platform with immune-activating potential.

The polymeric design of FPN_AI_—comprising PEG for biocompatibility and pH-responsive imidazole-containing polyaspartic acid—enabled efficient self-assembly, stable circulation, and controlled release. The observed increase in hydrodynamic diameter and altered zeta potential at acidic pH is consistent with enhanced protonation of responsive moieties, a validated strategy to achieve tumor-selective release of payloads [35]. Importantly, the incorporation of iICG not only enabled photodynamic activity after NIR irradiation but also demonstrated that the quenching and recovery of photoactivity by environmental triggers can be leveraged for spatial control of therapy, as previously reported in photoactivated nanomedicines [36]. Consistent with this concept, recent AIE-based nanomedicine platforms have shown that externally activated ROS generation can be used to amplify tumor cell death and reshape antitumor immunity, supporting the rationale for integrating photosensitizer-mediated ROS production into multifunctional nanotherapeutic systems [37, 38].

ABT-263 is a BH3 mimetic that antagonizes anti-apoptotic Bcl-2 family proteins, including Bcl-2, Bcl-xL, and Bcl-w, promoting intrinsic apoptosis [8, 39]. However, resistance mechanisms—particularly the upregulation of myeloid cell leukemia-1 (Mcl-1)—limit its efficacy in solid tumors [40]. The combination of photodynamically generated ROS with ABT-263 in FPN_AI_ may help attenuate this limitation by providing an additional pro-apoptotic stress and reducing compensatory anti-apoptotic signaling [41]. In our experiments, FPN_AI_ + NIR was associated with increased cleaved caspase-3 and reduced Bcl-2, Bcl-xL, and Mcl-1 levels, supporting the mechanistic complementarity between ABT-263-mediated intrinsic apoptosis and ICG-mediated PDT.

The enhanced ROS generation under acidic conditions upon NIR irradiation supports a more potent photodynamic effect, which not only contributes to direct cytotoxicity but also to immunogenic cell death (ICD). Tumor hypoxia should be considered when interpreting the PDT-related component of FPN_AI_ treatment, because singlet oxygen generation is oxygen-dependent and PDT can further consume local oxygen during the photodynamic process [42]. FPN_AI_ was not designed as an oxygen-supplying or hypoxia-relieving system; therefore, its ability to overcome hypoxia directly remains limited. However, the therapeutic activity of FPN_AI_ is not exclusively dependent on PDT, as ABT-263 directly promotes mitochondria-mediated apoptosis, while ICG-mediated ROS generation provides an additional pro-apoptotic stimulus after NIR irradiation. Future optimization may incorporate oxygen-generating or hypoxia-alleviating modules to further enhance PDT efficacy in hypoxic tumor regions.

Nanomedicine platforms designed to amplify PDT-induced ICD have demonstrated improved activation of dendritic cells and adaptive immunity, elevating long-term antitumor responses [43, 44]. The detection of multiple damage-associated molecular patterns (DAMPs), such as calreticulin exposure, HMGB1 release, ATP release, and upregulation of chemokines, supports the conclusion that FPN_AI_ induces bona fide ICD capable of bridging innate and adaptive immunity [45, 46]. The increased infiltration of CD8⁺ cytotoxic T lymphocytes and activated dendritic cells further indicates an immunostimulatory TME shift, a desirable outcome in “cold” tumors like HCC.

In vivo, FPNAI displayed enhanced accumulation in tumor tissue with prolonged retention compared with free ICG, consistent with active targeting via folate receptor interactions reported in other tumor systems [47]. This, combined with restored photodynamic activity, led to superior tumor growth inhibition in both xenograft and syngeneic HCC models. The minimal systemic toxicity observed across hematological and histopathological parameters suggests that the dual-responsive design effectively localizes therapeutic action while sparing normal tissues, addressing one of the key translational challenges for nanoparticle therapeutics [31, 41].

Despite these promising outcomes, several limitations should be acknowledged. First, while acute tumor responses and immune activation were documented, longitudinal studies evaluating durable immune memory and metastatic control are needed to determine the potential for long-term disease remission. Second, the heterogeneity of folate receptor expression in patient tumors may limit the universal applicability of the targeting strategy, and combinatorial targeting ligands might be required for broader clinical relevance. Third, although the current work demonstrates activation of ICD indicators, comprehensive profiling of systemic immune responses, including checkpoint modulation and suppressive myeloid populations, remains to be fully elucidated. Future optimization may also investigate the potential integration of immune checkpoint blockade with FPN_AI_ to further potentiate antitumor immunity, as suggested in recent nanomedicine-immunotherapy combinatorial strategies [30, 36].

In addition, although the present ABT-263/ICG regimen achieved significant antitumor efficacy with acceptable biosafety, the dose and ratio were selected to evaluate the therapeutic feasibility of FPN_AI_ rather than to define the optimal therapeutic window. This point is particularly relevant for nanoparticle-based combination therapy, because co-delivery platforms can help maintain drug exposure at relatively coordinated ratios and release rates, but the most effective ratio generally requires systematic optimization [48]. Future studies should include dose-ratio matrices, pharmacokinetic/pharmacodynamic analyses, and toxicity-guided optimization to identify the most effective and safest regimen for translational development.

## Conclusion

In this study, we constructed ABT-263-loaded bio-responsive nanoparticles with ICG for HCC-targeted therapy. The unique design of FPN_AI_ contains several advantages, including: (1) the polymer composed of hydrophobic PEG and hydrophilic PBLA can overcome the limitation of single material carrying drugs, and can achieve the goal of controlled release of drugs; (2) FR is specially enriched on cell membrane of tumor cells, so FPN_AI_ loading FA can specific accumulate around tumor cells through circulation system. In light of this, FPN_AI_ can achieve the goal of improving the concentration of ABT-263 at the site of HCC which is the main factors limiting the effect of ABT-263 in treating HCC, and of accurately treating HCC without causing systemic adverse events; (3) ICG mediated PDT can kill HCC cells in cooperation with ABT-263 once giving NIR irradiation, improving the clinical value of FPN_AI_; and (4) FPN_AI_ treatment can induce ICD in HCC. In summary, FPN_AI_ combing NIR is highly translatable as a potential method for the treatment of HCC.

## Supplementary Information

Supplementary material 1.

Supplementary material 2.

Supplementary material 3.

Supplementary material 4.

Supplementary material 5.

Supplementary material 6.

Supplementary material 7.

Supplementary material 8.

Supplementary material 9.

Supplementary material 10.

Supplementary material 11.

Supplementary material 12.

Supplementary material 13.

## Data Availability

All data generated or analysed during this study are included in this published article and its supplementary information files.
