# Supplementary material for: Navitoclax-encapsulated dual pH-responsive copolymeric nanoparticles improves hepatocellular carcinoma treatment with induction of apoptosis and immune activation
Source: J Nanobiotechnology. 2026 Jul 14;24:944. doi: 10.1186/s12951-026-04733-3 (PMC13651130; doi:10.1186/s12951-026-04733-3)
Supplement: Supplementary file 1 [file 12951_2026_4733_MOESM11_ESM.pdf]

Fig.3 g

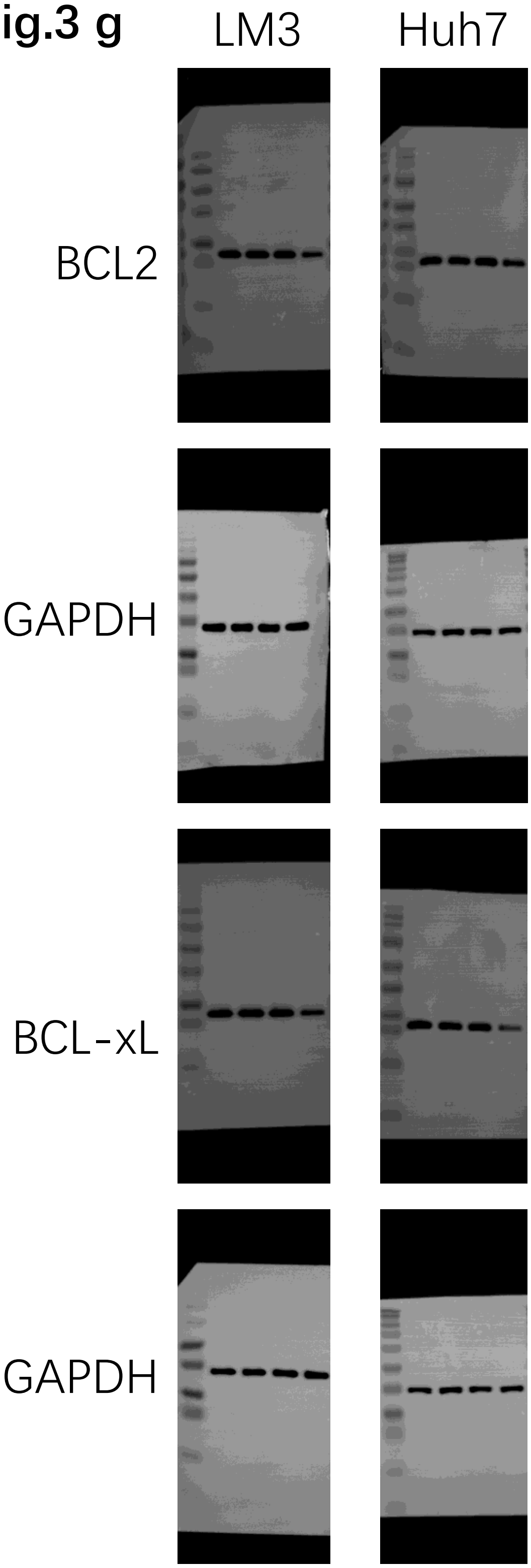
LM3
Huh7
BCL2
GAPDH
BCL-xL
GAPDH

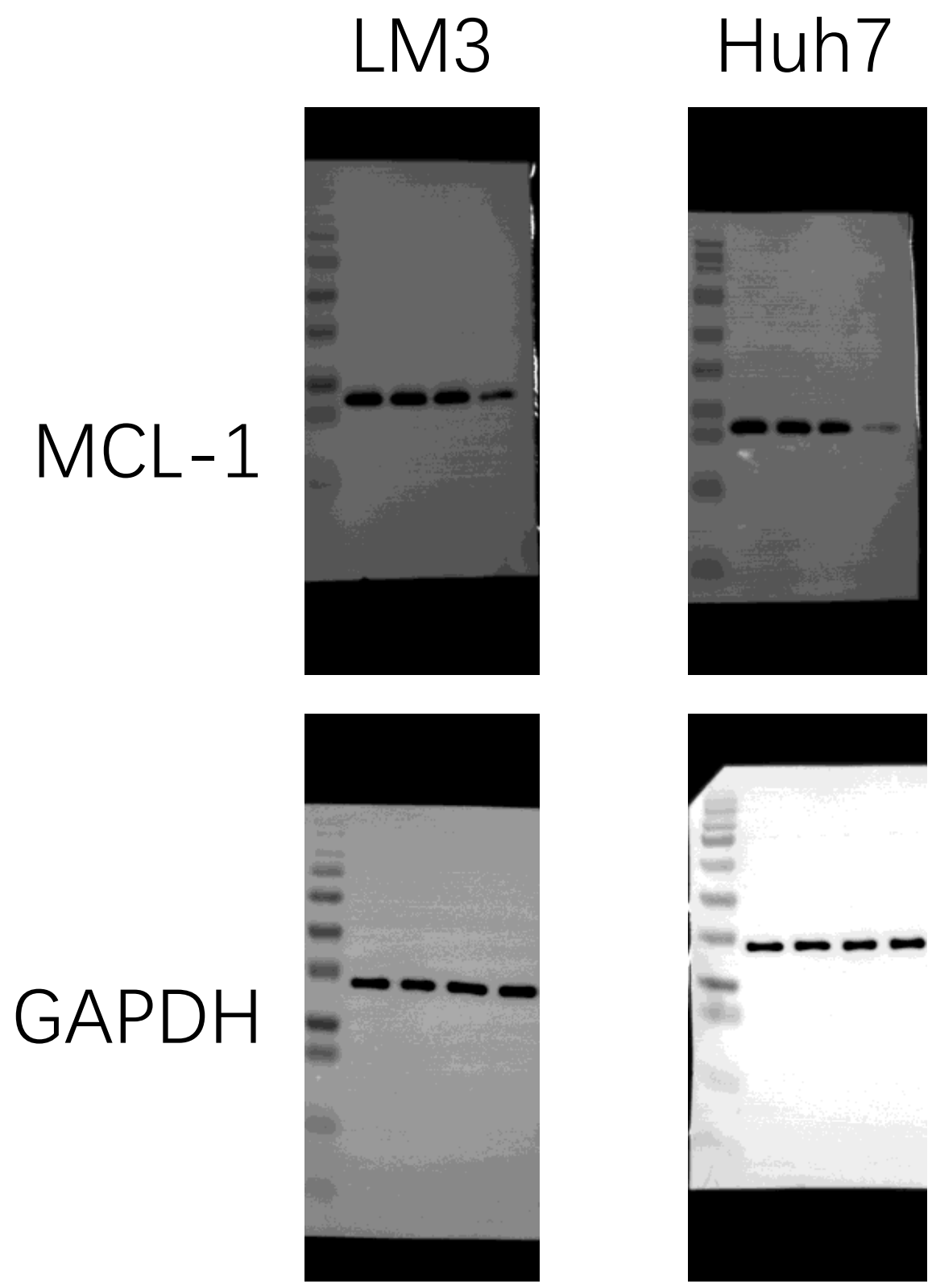
LM3
Huh7
MCL-1
GAPDH

Fig.3 j

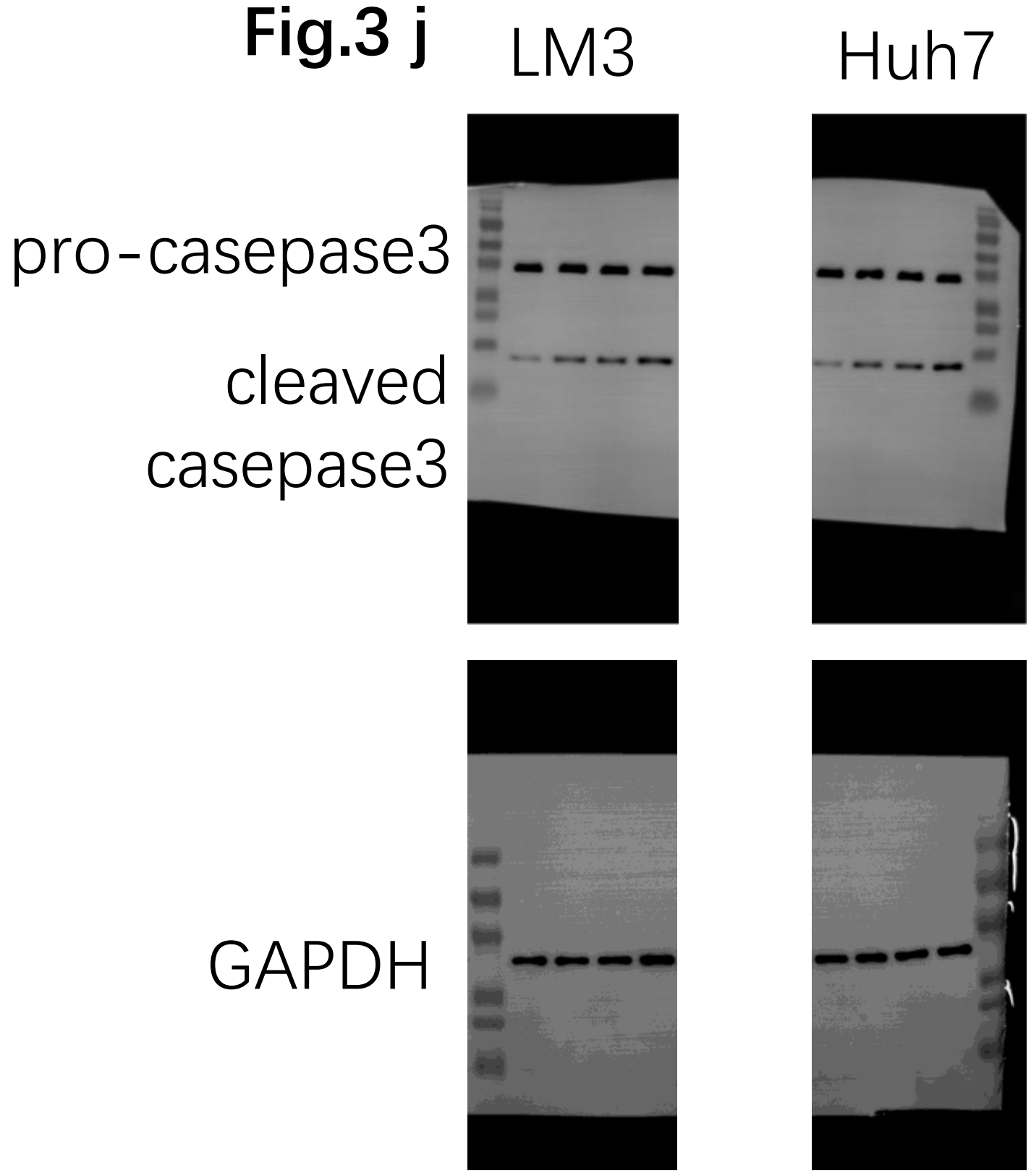
LM3
Huh7
pro-casepase3
cleaved
casepase3
GAPDH
