# Supplementary material for: Navitoclax-encapsulated dual pH-responsive copolymeric nanoparticles improves hepatocellular carcinoma treatment with induction of apoptosis and immune activation
Source: J Nanobiotechnology. 2026 Jul 14;24:944. doi: 10.1186/s12951-026-04733-3 (PMC13651130; doi:10.1186/s12951-026-04733-3)

Fig.4 e

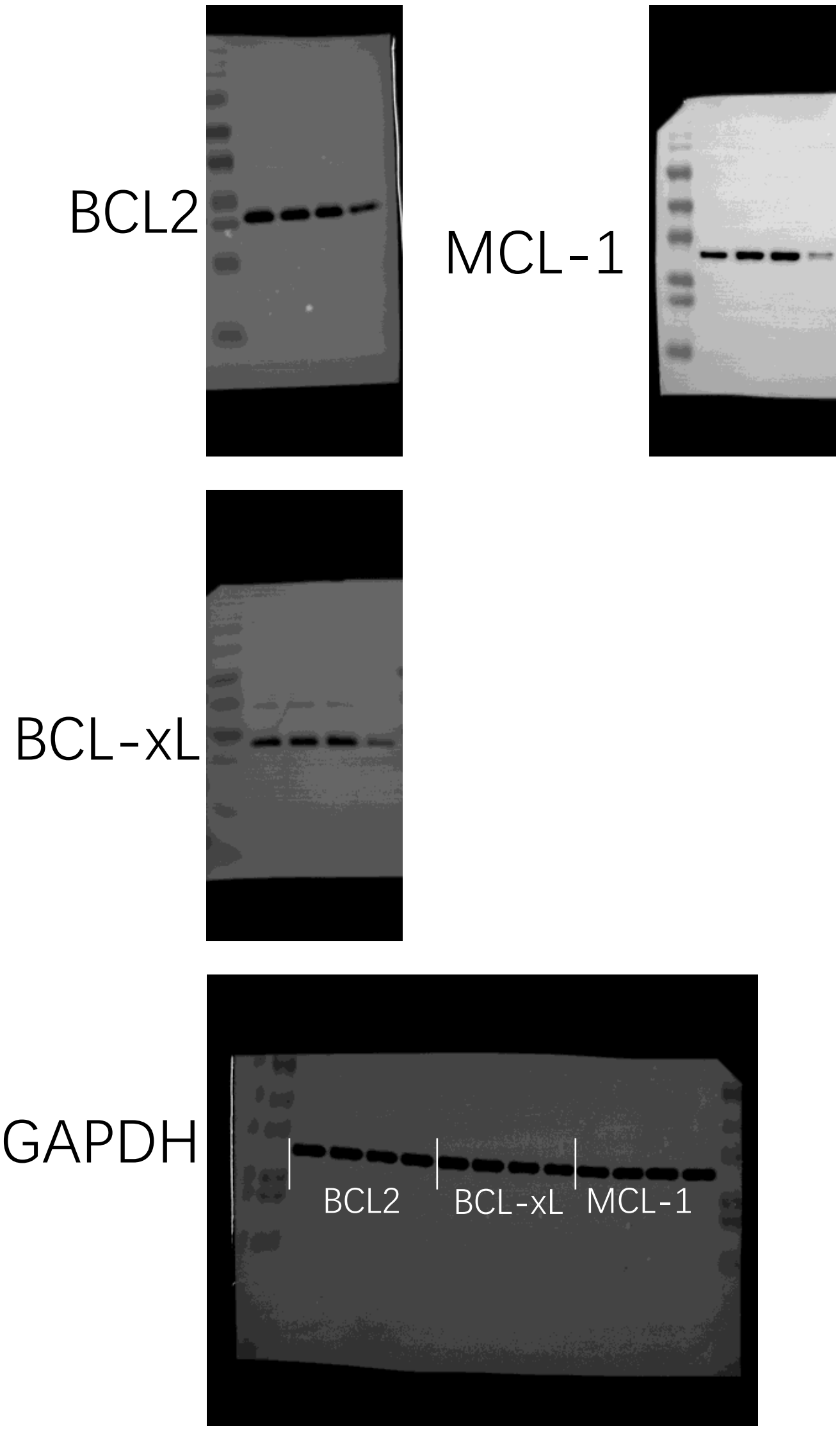

Fig.4 m

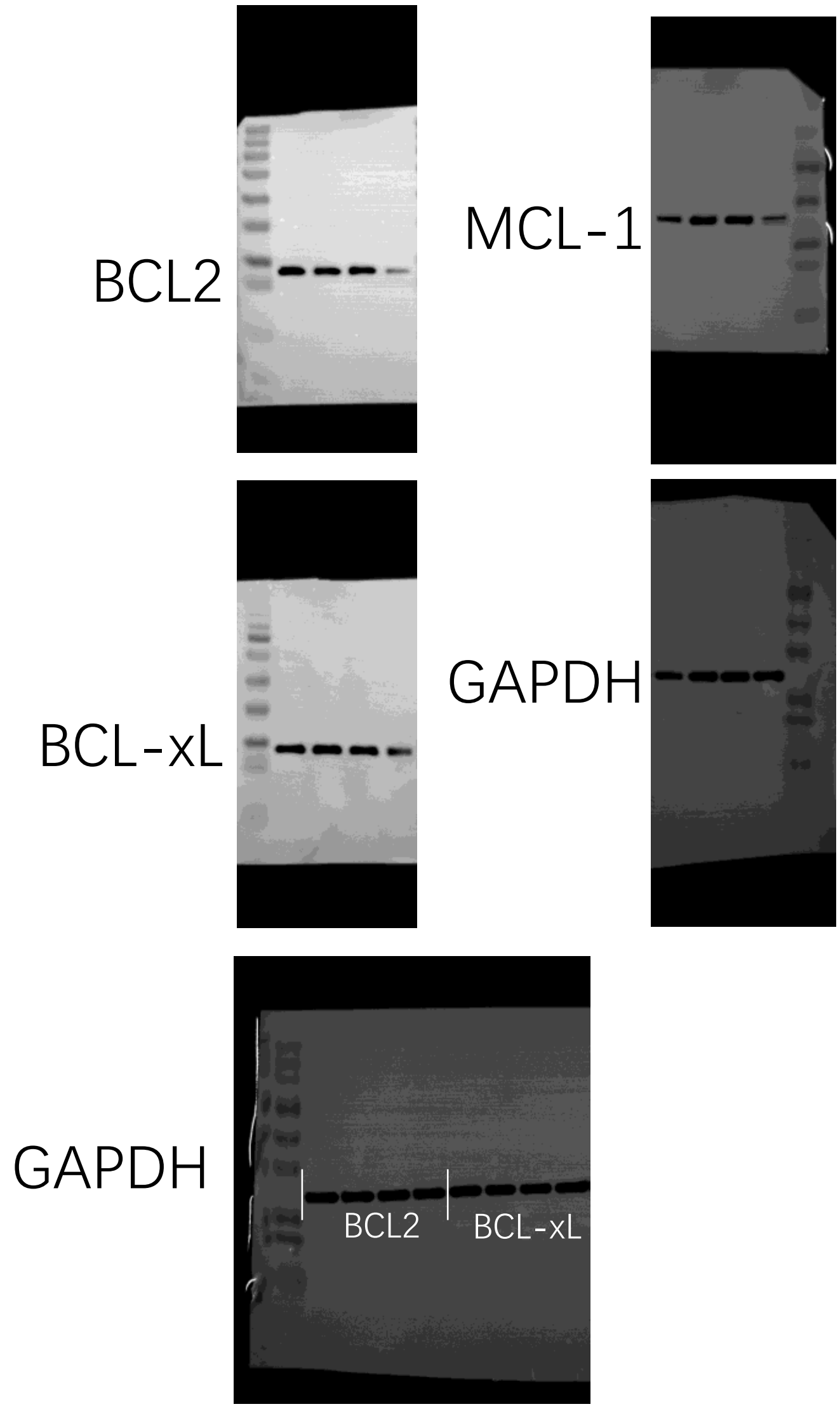

Fig.4 g

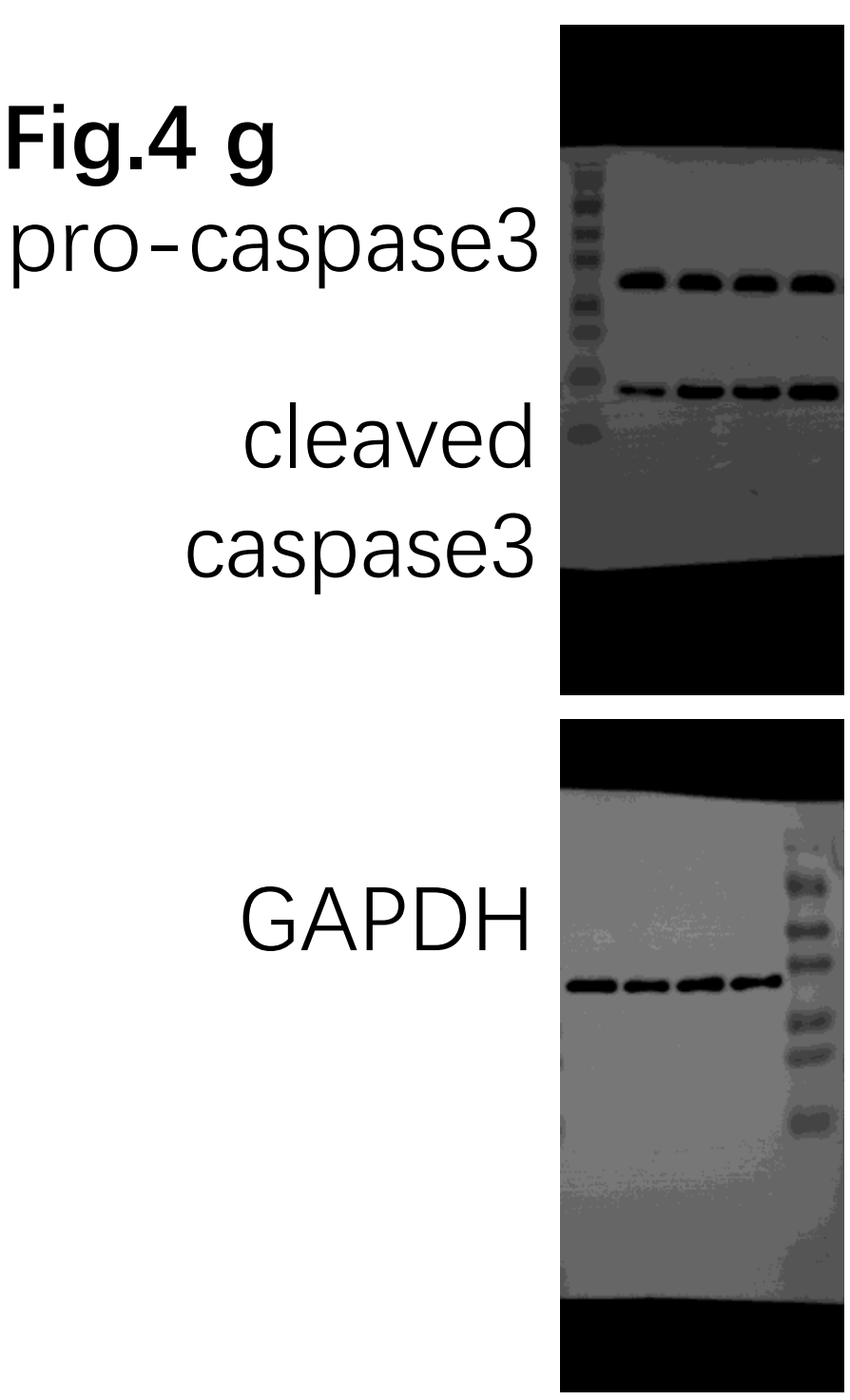

Fig.4 o

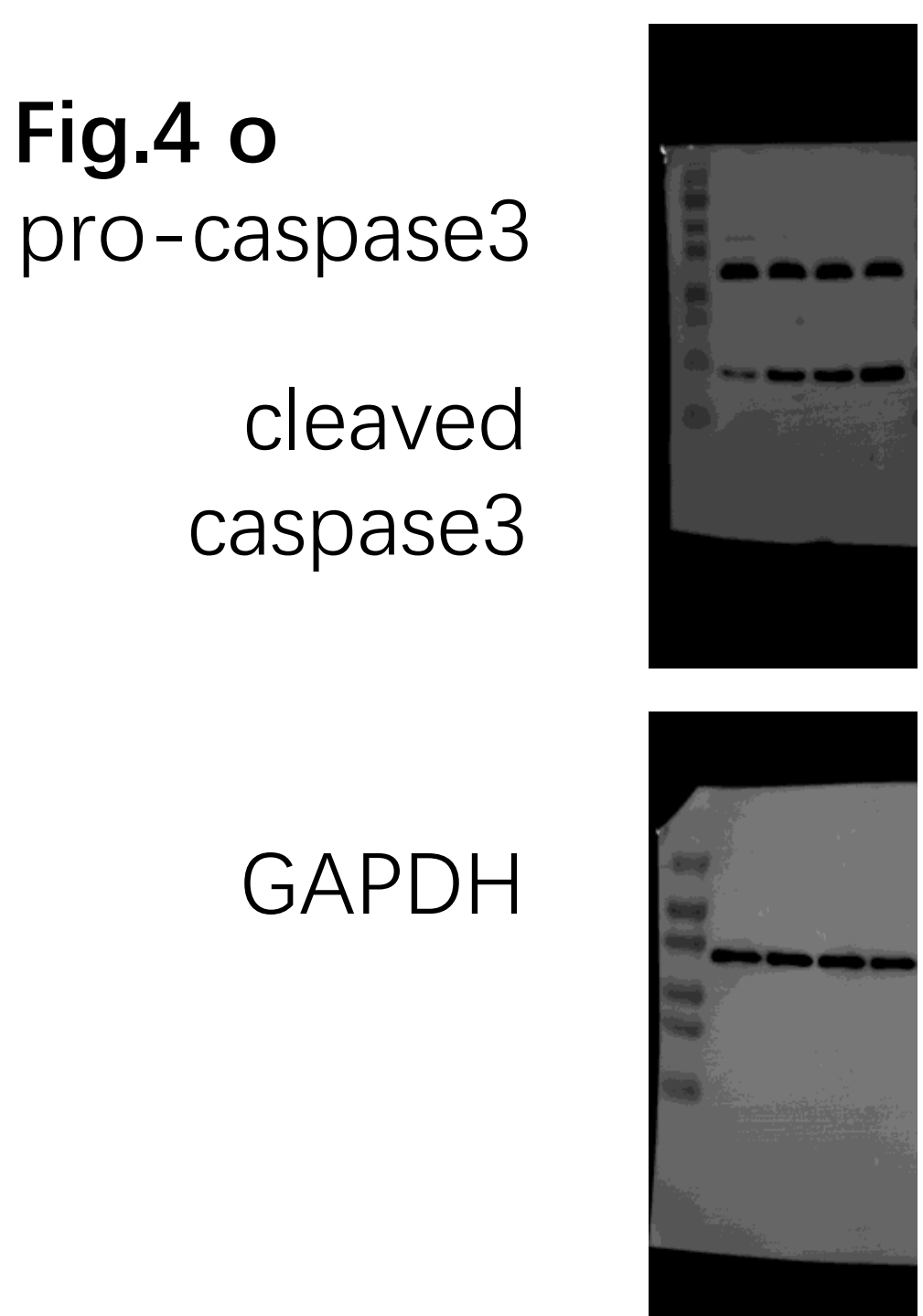

Supplement: Supplementary file 2 [file 12951_2026_4733_MOESM12_ESM.pdf]
