# Supplementary material for: Navitoclax-encapsulated dual pH-responsive copolymeric nanoparticles improves hepatocellular carcinoma treatment with induction of apoptosis and immune activation
Source: J Nanobiotechnology. 2026 Jul 14;24:944. doi: 10.1186/s12951-026-04733-3 (PMC13651130; doi:10.1186/s12951-026-04733-3)
Supplement: Supplementary file 3 [file 12951_2026_4733_MOESM13_ESM.pdf]

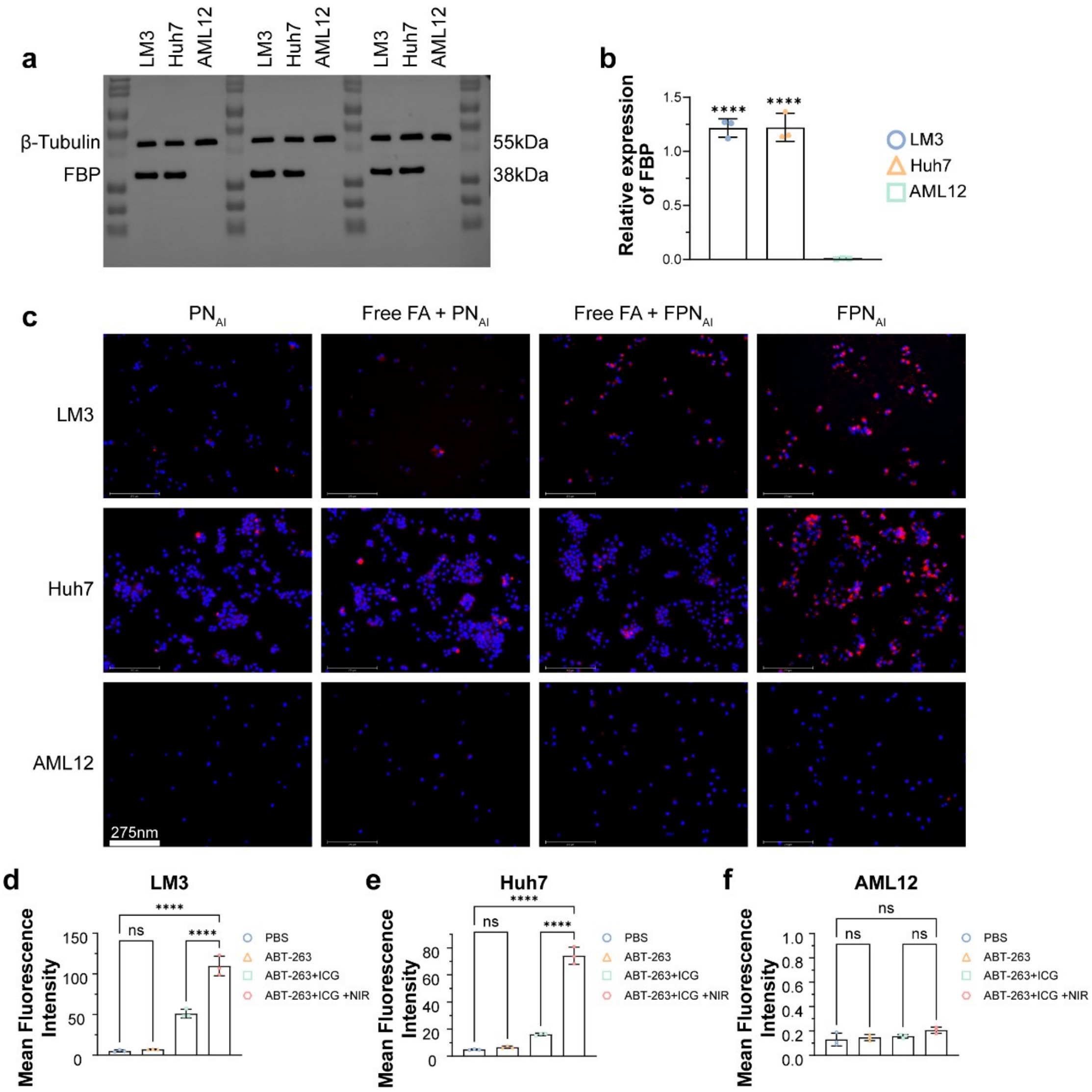

Figure S11. Folate receptor expression and target-specific uptake of FPN_{AI}.

(a) Western blot analysis of FBP expression in LM3, Huh7, and AML12 cells. β-Tubulin was used as the loading control. (b) Quantification of FBP protein expression normalized to β-tubulin. (c) Representative fluorescence images showing the cellular uptake of PN_{AI} and FPN_{AI} in LM3, Huh7, and AML12 cells with or without free FA preincubation. Red fluorescence indicates ICG signal, and blue fluorescence indicates DAPI-stained nuclei. (d–f) Quantification of mean fluorescence intensity in LM3, Huh7, and AML12 cells. Data are presented as mean ± SD, n = 3. Statistical analysis was performed using one-way ANOVA with multiple comparisons. ns, not significant; ****$P < 0.0001$.

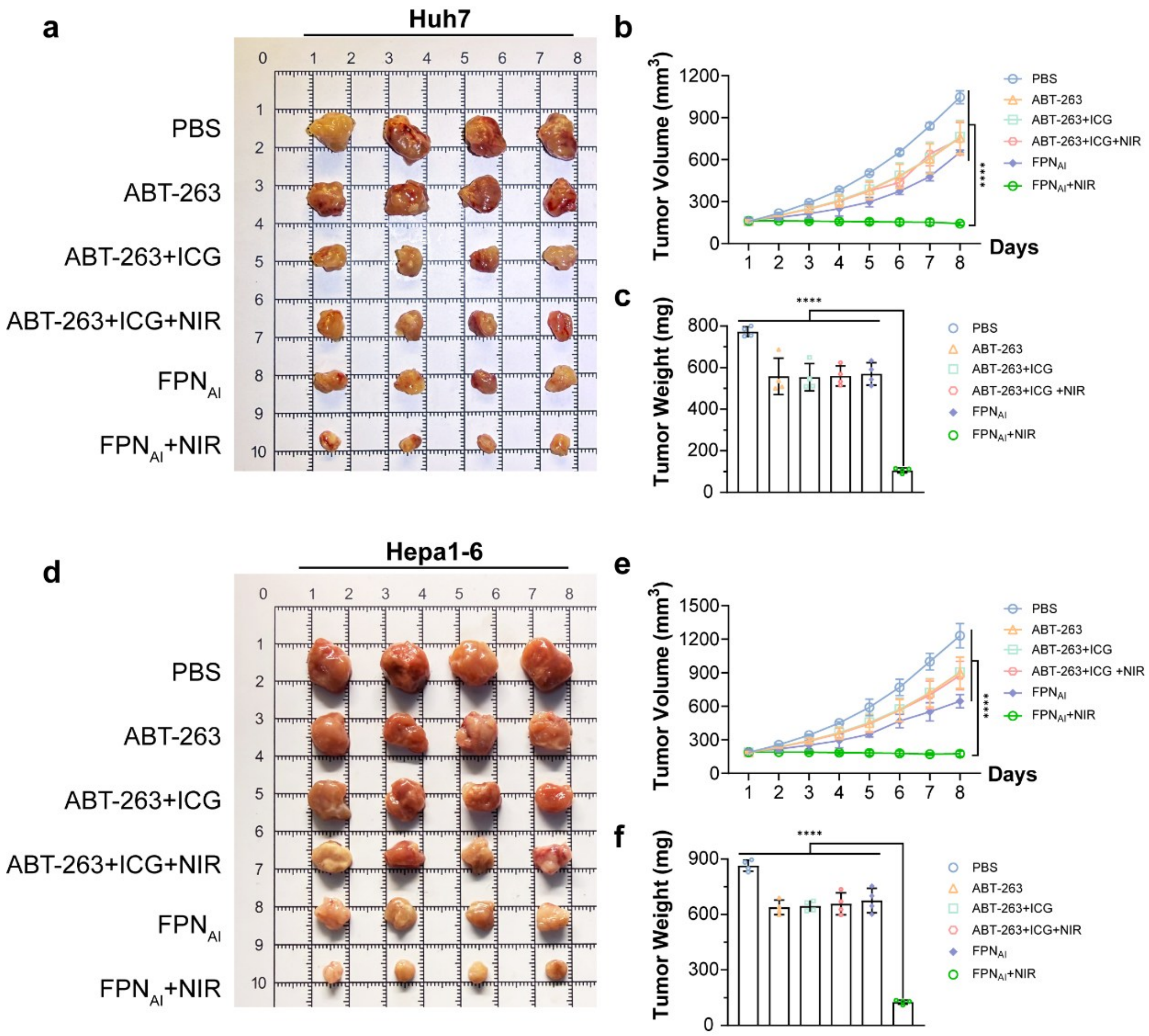

Figure S12. Additional in vivo comparison of FPNAI with free drug co-administration.

(a) Gross tumor images from Huh7 xenograft-bearing mice treated with PBS, free ABT-263, free ABT-263 plus free ICG, free ABT-263 plus free ICG with NIR irradiation, FPN_{AI}, or FPN_{AI} with NIR irradiation. (b) Tumor volume curves of Huh7 xenograft-bearing mice during treatment. (c) Endpoint tumor weights of Huh7 xenografts. (d) Gross tumor images from Hepa1-6 tumor-bearing mice treated with the indicated formulations. (e) Tumor volume curves of Hepa1-6 tumor-bearing mice during treatment. (f) Endpoint tumor weights of Hepa1-6 tumors. Data are presented as mean ± SD, n = 4 mice per group. Statistical analysis was performed using one-way ANOVA with multiple comparisons or two-way ANOVA for tumor growth curves. **$P < 0.01$, ****$P < 0.0001$.

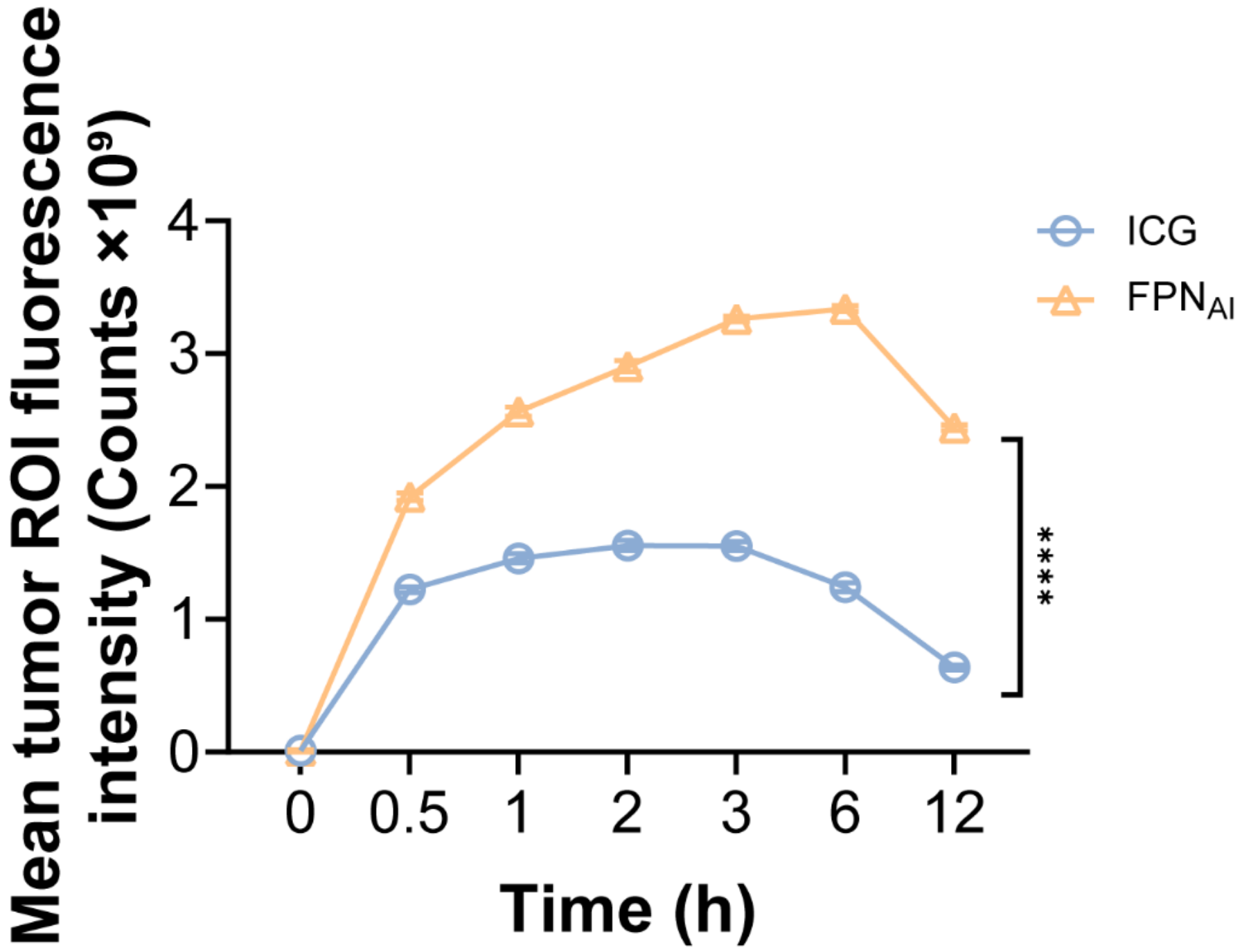

Figure S13. Quantitative analysis of tumor-region fluorescence intensity in vivo.

ROI-based quantification of tumor fluorescence intensity in HCC-bearing mice after intravenous injection of free ICG or FPN_{AI} at an equivalent ICG dose. Tumor ROIs were defined according to the green dashed circles shown in Figure 6b. Data are presented as mean fluorescence intensity in the tumor ROI. ****$P < 0.0001$. Data are presented as mean ± SD, n = 3.

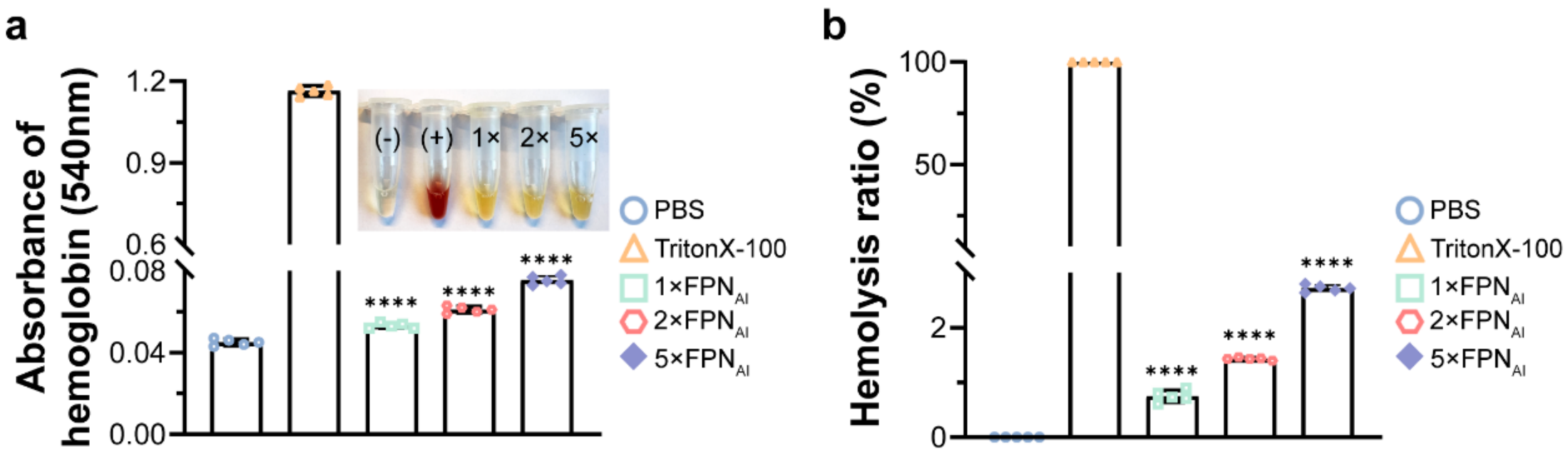

Figure S14. Hemolysis assay of FPNAI.

(a) Absorbance of released hemoglobin at 540 nm after incubation of red blood cells with PBS, Triton X-100, or FPN_{AI} at 1×, 2×, and 5× concentrations. The inset shows representative images of the supernatants after centrifugation. (b) Hemolysis rates calculated from the absorbance values at 540 nm. PBS was used as the negative control and Triton X-100 as the positive control. Data are presented as mean ± SD, n = 5. ****$P < 0.0001$ vs. Triton X-100.
